# Antarctic ice sheet discharge driven by atmosphere-ocean feedbacks at the Last Glacial Termination

**DOI:** 10.1038/srep39979

**Published:** 2017-01-05

**Authors:** C. J. Fogwill, C. S. M. Turney, N. R. Golledge, D. M. Etheridge, M. Rubino, D. P. Thornton, A. Baker, J. Woodward, K. Winter, T. D. van Ommen, A. D. Moy, M. A. J. Curran, S. M. Davies, M. E. Weber, M. I. Bird, N. C. Munksgaard, L. Menviel, C. M. Rootes, B. Ellis, H. Millman, J. Vohra, A. Rivera, A. Cooper

**Affiliations:** 1 PANGEA Research Centre, University of New South Wales, 2052, Australia; 2 Climate Change Research Centre, School of Biological Earth and Environmental Sciences, University of New South Wales, 2052, Australia; 3 Antarctic Research Centre, Victoria University of Wellington, Wellington 6140, New Zealand; 4 GNS Science, Avalon, Lower Hutt, New Zealand; 5 CSIRO Climate Science Centre, Oceans and Atmosphere, Aspendale, Victoria, 3195 Australia; 6 Dipartimento di Matematica e Fisica, Università della Campania - Luigi Vanvitelli, viale Lincoln, 5-81100 Caserta, Italy; 7 Department of Geography, Faculty of Engineering and Environment, Northumbria University, Newcastle upon Tyne, NE1 8ST, United Kingdom; 8 Australian Antarctic Division, 203 Channel Highway, Kingston, Tasmania 7050, Australia; 9 Antarctic Climate & Ecosystems Cooperative Research Centre, University of Tasmania, Private Bag 80, Hobart, Tasmania 7001, Australia; 10 Department of Geography, College of Science, Swansea University, Swansea, United Kingdom; 11 Department of Earth Sciences, University of Cambridge, Drummond Street, Cambridge, United Kingdom; 12 Steinmann Institute, University of Bonn, Poppelsdorfer Schloss, Bonn, Germany; 13 Centre for Tropical Environmental and Sustainability Science, College of Science and Engineering, James Cook University, Cairns, Australia; 14 Research Institute for the Environment and Livelihoods, Charles Darwin University, Australia; 15 Department of Geography, University of Sheffield, United Kingdom; 16 Research School of Earth Sciences, Australian National University, Canberra, Australia; 17 Glaciology and Climate Change Laboratory, Centro de Estudios Cientficos, Valdivia, Arturo Prat 514, Chile; 18 Department of Geography, University of Chile, Santiago, Chile; 19 Australian Centre for Ancient DNA, University of Adelaide, 5005, Australia

## Abstract

Reconstructing the dynamic response of the Antarctic ice sheets to warming during the Last Glacial Termination (LGT; 18,000–11,650 yrs ago) allows us to disentangle ice-climate feedbacks that are key to improving future projections. Whilst the sequence of events during this period is reasonably well-known, relatively poor chronological control has precluded precise alignment of ice, atmospheric and marine records, making it difficult to assess relationships between Antarctic ice-sheet (AIS) dynamics, climate change and sea level. Here we present results from a highly-resolved ‘horizontal ice core’ from the Weddell Sea Embayment, which records millennial-scale AIS dynamics across this extensive region. Counterintuitively, we find AIS mass-loss across the full duration of the Antarctic Cold Reversal (ACR; 14,600–12,700 yrs ago), with stabilisation during the subsequent millennia of atmospheric warming. Earth-system and ice-sheet modelling suggests these contrasting trends were likely Antarctic-wide, sustained by feedbacks amplified by the delivery of Circumpolar Deep Water onto the continental shelf. Given the anti-phase relationship between inter-hemispheric climate trends across the LGT our findings demonstrate that Southern Ocean-AIS feedbacks were controlled by global atmospheric teleconnections. With increasing stratification of the Southern Ocean and intensification of mid-latitude westerly winds today, such teleconnections could amplify AIS mass loss and accelerate global sea-level rise.

Understanding centennial to millennial-scale variability of the Earth’s ice sheets is key to gaining insights into ice sheet-climate feedbacks^1^,^2^, and quantifying their contribution to past and future environmental change^3^,^4^. This is important, as despite mounting evidence of significant changes in AIS dynamics^5^, Southern Ocean^6^, and atmospheric circulation^7^, current projections of global mean sea level (GMSL) imply only moderate increases by the end of the twenty-first century^3^. These projections, however, do not fully include ice-sheet-ocean dynamic feedbacks which are believed to have triggered rapid continental ice-sheet retreat and driven periods of abrupt sea-level rise during the geological past^2^,^8^. The LGT offers a potential process analogue for future climate trends, characterised by multi-millennial global warming, poleward migrating and strengthening westerly winds^9^, and increasing atmospheric carbon dioxide levels^10^, similar in magnitude to future projections^4^.

During the LGT, long-term warming was interrupted by the ~2,000-year duration cold event across the mid to high latitude Southern Hemisphere, known as the Antarctic Cold Reversal^11^,^12^, which was associated with a ~35 m GMSL rise. Within the ACR, Meltwater Pulse 1A (MWP-1A) forms a prominent abrupt rise in sea level of ~16 m (14,700–14,300 years or 14.7–14.3 ka) that has been a major focus of previous studies, and which was coincident with a period of enhanced iceberg flux in the Southern Ocean^2^. However, the actual contribution of the AIS during this period remains unclear^13^,^14^ due to the paucity of geological records capable of resolving ice-sheet volume changes in such a dynamic contemporary ice sheet setting^15^, and the difficulties in precisely aligning the chronologies of marine and terrestrial sequences^2^,^11^. While the contribution of AIS to GMSL rise during MWP-1A range from ‘high-end’ scenarios (>10 m contributing over half of the total GMSL rise), to ‘low-end’ (scenarios with little to no contribution), the AIS input (if any) during the ACR and the subsequent period of sustained Southern Hemisphere warming remains debated^14^,^16^,^17^. Crucially, the role of the AIS in global climate-ocean dynamics during the LGT remain uncertain^11^,^18^. An improved understanding of the links between AIS stability and ice-ocean-climate feedbacks throughout the LGT (i.e. not just MWP-1A), and its relationship to Northern Hemisphere changes, is therefore critical for improving projections of sea-level rise^3^,^4^ and understanding ice-sheet-climate feedbacks^2^,^17^ in detail.

Here we take a novel approach that investigates a new 800 m long ‘horizontal ice core’ that captures a unique record of ice-sheet dynamics and climate across the Weddell Sea Embayment (WSE)^19^, a region which today drains more than one-fifth of the ice-mass of continental Antarctica, including sectors of the East and West Antarctic ice sheets and the Antarctic Peninsula (Fig. 1A).

## Results

**Figure 1:**
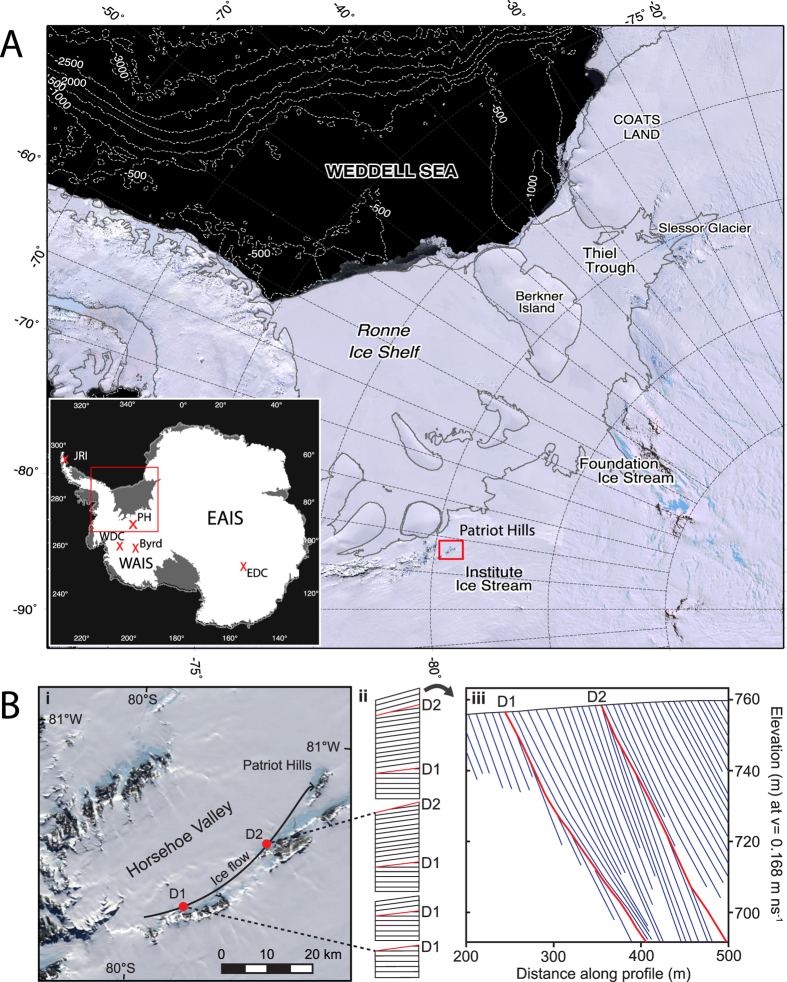
(**A**) Location map of Weddell Sea Embayment (WSE)^36^ and the major ice streams, with the location of Patriot Hills in the Ellsworth Mountains. Lower left. Inset map of Antarctica, with locations of the Patriot Hills (PH), WAIS Divide (WDC), Byrd, James Ross Island (JRI) and EPICA Dome C (EDC) ice cores, and the East Antarctic Ice Sheet (EAIS) and West Antarctic Ice Sheet (WAIS). (**B**) (i). Moderate Resolution Imaging Spectroradiometer (MODIS) mosaic^36^ showing inferred ice flow path from the head of Horseshoe Valley to Patriot Hills, where discontinuities D1 and D2 formed as a result of Blue Ice Area wind scour in front of Liberty and Marble Hills respectively^20^, (ii) schematic stratigraphic succession, indicating ice accumulation punctuated by two periods of erosion (D1 and D2; red lines). The uppermost panel of ii represents the observed stratigraphic sequence at the Patriot Hills BIA as seen in (iii), the full GPR stratigraphic sequence at Patriot Hills BIA, where red lines indicate erosional events D1 and D2. Adapted from Winter *et al*.^20^.

**Figure 2:**
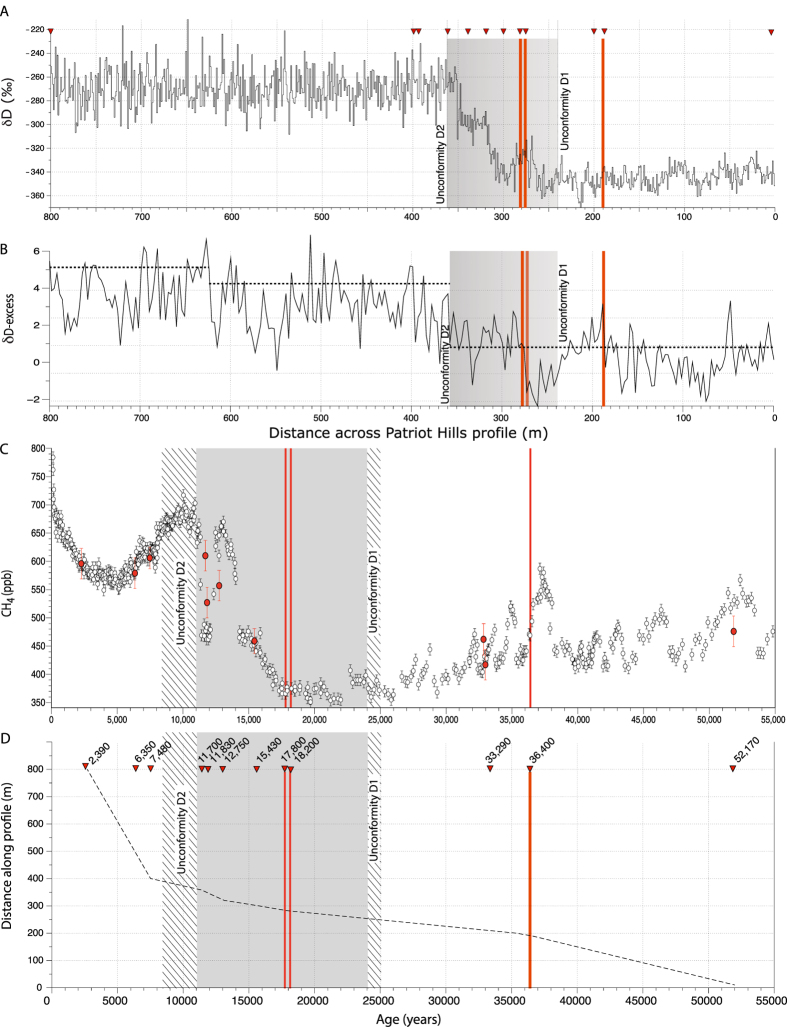
(**A**) Full δD isotopic profile from the Patriot Hills BIA with chronological age ties (red triangles) highlighted. The red bars highlight the location of volcanic horizons at 17.8 ka, 18.2 ka and 36.4 ka, as recorded in other Antarctic ice core records (Supplementary Information). The grey bars highlight the area of the profile between the unconformities at 247 m (D1) and 360 m (D2) between which the GPR survey demonstrates clear dipping reflectors or isochrons across the profile (see Fig. 1B). (**B**) δD-excess across profile; dashed horizontal lines denote potential regime shifts across the profile at 99% confidence^37^. (**C**) CH_4_ concentrations from ice extracted from the Patriot Hills profile (filled red circles) plotted against EPICA Dome C (EDC)^34^,^35^ (open white circles), with the approximate timings of the unconformities outlined by the hatched areas and 1σ uncertainty. (**D**) Age-depth model based upon chronological control ties between ~2.5 ka (2,540 years) and ~52 ka (52,170 years) from volcanic ‘tephra’ horizons and most-likely age as derived from multiple trace gas comparison to published records (CH_4_, CO_2_, N_2_O; see Methods and Supplementary Information). Note: the timing of the onset of ice accumulation after D2 in Patriot Hills is a conservative estimate and with future trace gas dating may be younger than that presented here.

We report results from an exposed ancient blue ice area (BIA) outcropping alongside Patriot Hills in Horseshoe Valley (Fig. 1A), a locally sourced compound glacier that is buttressed by, but ultimately coalesces with the Institute Ice Stream close to the contemporary grounding line of the AIS^20^, making the site highly sensitive to elevation changes across the broader WSE region^21^. Geochemically identified volcanic (tephra) horizons along with multiple trace gas species (CO_2_, CH_4_ and N_2_O) provide key age tie points across the profile (Fig. 2; Methods and Supplementary Information), and demonstrate that the profile spans from ~2.5 to 50 ka, with two unconformities (discontinuities D1 and D2; Fig. 1B) that mark the build up to, and deglaciation from, the Last Glacial Maximum (see Methods)^20^. The conformable BIA layers or ‘isochrons’ between these two unconformities span ~11 to ~23 ka, capturing a unique highly-resolved record of ice-sheet dynamics across the LGT in an area of exceptionally slow-moving ice^20^ (Fig. 1B).

**Figure 3:**
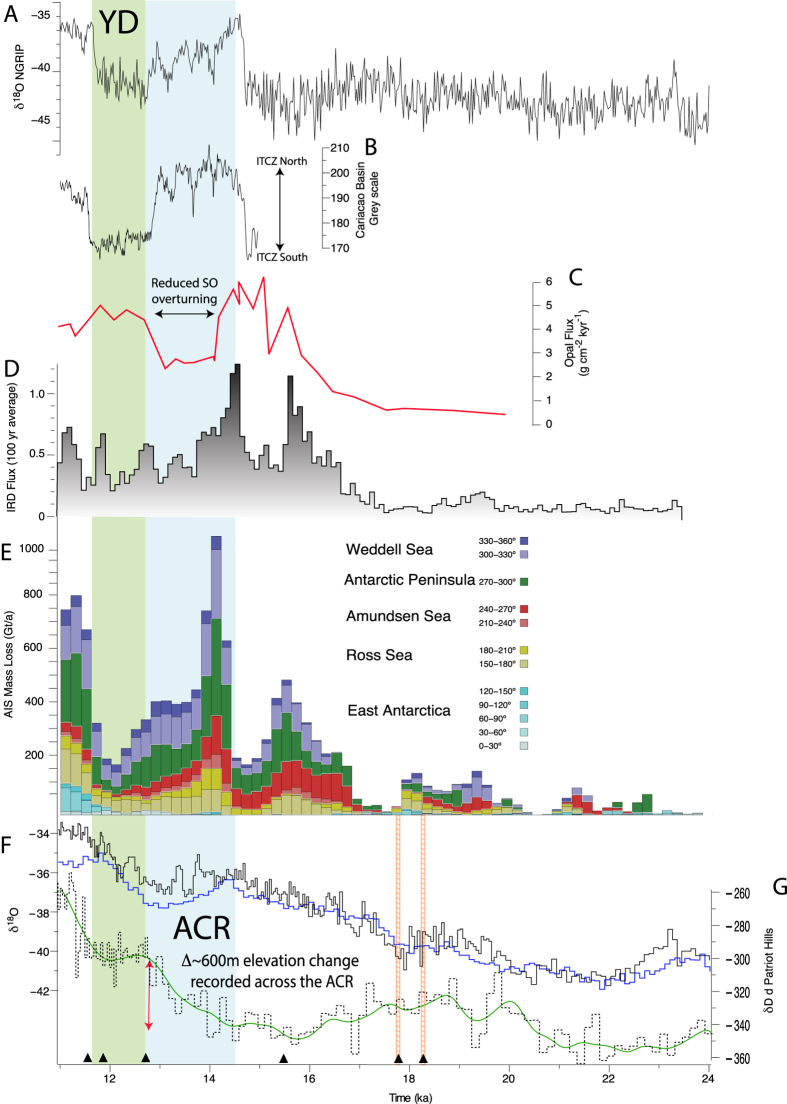
Inter-comparison of deglacial elevation changes from Patriot Hills BIA with modelled and empirical records. (**A**) δ^18^O from NGRIP (GICC05 chronology)^38^. (**B**) Cariaco Basin grey scale, a measure of latitudinal changes in the trade winds associated with the ITCZ^12^. (**C**) Southern Ocean opal flux^9^. (**D**) Iceberg-rafted debris flux (IRD; 100-year average) relative to Holocene average from the Scotia Sea^2^. (**E**) Modelled sector-wide AIS mass loss^17^. (**F**) Byrd δ^18^O (blue) (synchronised to GISP2 chronology) isotopic record^39^ and WAIS Divide Core δ^18^O (WD2014 chronology) (black)^23^ correlated with the volcanic horizons at 18 ka and 18.2 ka. (**G**) δD isotope profile (black dashed line), with 2-point moving average (green solid line). The red arrow highlights the apparent ∼600 m ice-sheet surface elevation change across the WSE estimated from the δD isotopic changes recorded during the ACR from the Patriot Hills BIA. Vertical boxes identify the periods defined by the Antarctic Cold Reversal (ACR) (blue) and the Younger Dryas chronozone (YD) (green). The black triangles represent the age tie points (derived from geochemically identified volcanic horizons and trace gases) in this section of the Patriot Hills BIA.

The water stable isotope deuterium (δD) from the Patriot Hills BIA identifies a two-stepped change in values during the LGT, with a ~39‰ increase recorded across the ACR between ~14.7–12.7 ka, followed by a millennial-duration isotopic plateau (~12.7–11.7 ka) (Fig. 3G). Using a regionally applied δD–temperature relationship of 6.4 ± 1.3‰ per °C^22^, the Patriot Hills record implies an increase in annual temperature of ~6 °C across the ACR with little to no subsequent change up to ~11.7 ka; comparable trends are also recorded in the δ^18^O profile (SI Figure S6). The deuterium excess values demonstrate there is no significant regime shift across the profile during the LGT, suggesting no change in precipitation source, or the sign of the isotope-temperature relationship (Fig. 2). These apparent temperature changes are in marked contrast to regional climate records across continental Antarctica^23^ and the Antarctic Peninsula^22^ that show a clear plateau/reversal in the warming trend during the ACR (Fig. 3). Given the isolated nature of Horseshoe Valley both during contemporary times and at the LGT^19^,^20^, and the buttressing effect of the AIS on ice flow from the valley (Fig. 1), we interpret the isotopic trend captured in the Patriot Hills BIA as the result of ice-sheet elevation changes due to mass loss across the broader WSE^21^ (see Methods). Thus, increasing δD (and δ^18^O; Supplementary Information) water isotope values across the ACR and the apparent local warming can only reflect regional ice-sheet draw down. The isotopic profile that followed (~12.7–11.7 ka) appears to reflect a stabilisation of ice-sheet elevation for approximately a millennium.

The marked change in δD captured in the record across the ACR implies an ice-sheet surface elevation decrease of ~615 m across Horseshoe Valley (at a rate of ~0.4 m/a), assuming an atmospheric lapse rate of 10 °C/km^24^. This rate of change across the WSE is similar to that inferred from terrestrial cosmogenic isotope studies of mid-Holocene glacier thinning in the WSE^25^, and lower than recorded in the Amundsen Sea sector of the West Antarctic in recent decades^5^. Importantly, the projected elevation changes represent absolute minima. If the effects of regional ACR cooling and potential glacial isostatic rebound are included this value would exceed ~800 m. The period of mass loss we identify in the WSE parallels a period of enhanced iceberg-rafted debris flux as recorded in marine sediments from the Scotia Sea (Fig. 3D)^2^, strongly indicating enhanced AIS iceberg discharge was sustained across the ACR (that included but was not limited to MWP-1A). Importantly, during the subsequent period, modelled ice-sheet outputs (see Methods) suggest a marked reduction in AIS drawdown between 12.7 and 11.7 ka (Fig. 3E and Supplementary Information), consistent within the uncertainties of the Patriot Hills BIA chronology (Supplementary Information) of invariant stable isotope values following the ACR (Fig. 3G).

## Discussion

**Figure 4:**
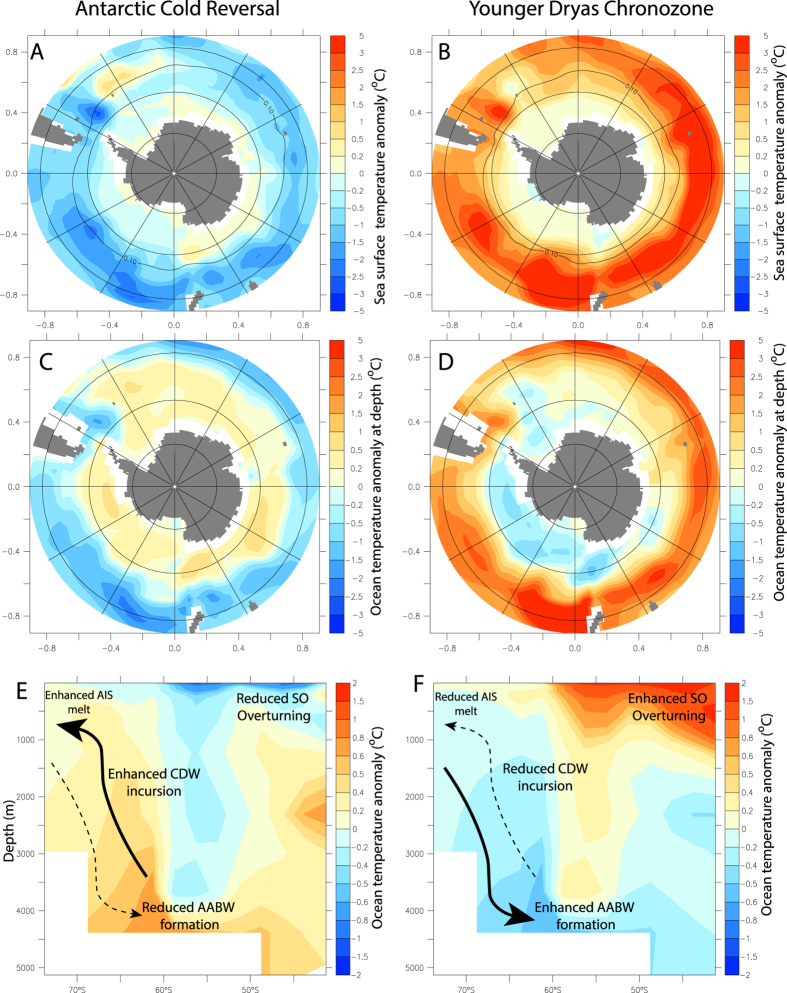
LOVECLIM transient model simulations of Southern Ocean fresh water forcing showing temperature anomalies (°C) for the ACR (14 ka minus 15 ka; left-hand panels)^17^,^18^, and subsequent surface warming during the Younger Dryas chronozone (12 ka minus 14 ka right-hand panels), with sea surface temperatures and 0.1 m sea ice contour (**A**,**B**), ocean temperature anomalies at depth (**C**,**D**, averaged over 484–694 m), and ocean temperature anomalies across the Weddell Sea (60°W to 15°W) (**E**,**F**) (constructed using ferret http://ferret.pmel.noaa.gov/Ferret/).

The regional climate and sea-level fingerprint of the ACR across the mid- to high-latitude Southern Hemisphere is difficult to reconcile. Recent modelling studies have demonstrated that it is possible to reconstruct the spatial pattern without substantial fresh water forcing in the Southern Ocean^11^,^14^. However, our modelling, together with the results of previous studies^2^,^13^,^18^, suggests that a significant fresh water input into the Southern Ocean provides a potential trigger for the ACR signal, a hypothesis supported by our field data. Our inferred decoupling of ice-sheet elevation from air temperature across the LGT implies ocean forcing was a primary driver of Antarctic-wide ice-sheet dynamics through this period. Independent ice-sheet modelling experiments, driven by transient Earth System Model (LOVECLIM) outputs that include fresh water hosing in the Ross and Weddell seas^17^ (see Supplementary Information), predict similar changes in ice-sheet geometry and ice-flow dynamics (Figs 3 and 4 and Supplementary Information). The modelled increase in freshwater flux strongly suggests the drawdown of the AIS during the ACR was sustained by a positive feedback operating within the Southern Ocean. Crucially, we find a freshening of surface waters leads to a weakening of Southern Ocean overturning, resulting in reduced Antarctic Bottom Water (AABW) formation, enhanced stratification and sea-ice expansion^17^ (Fig. 4). The increased delivery of relatively warm Circumpolar Deep Water (CDW)^26^ onto the continental shelf close to the grounding line of the AIS thermally erodes marine-based ice, maintaining a positive ice-ocean feedback (Fig. 4)^17^. High resolution ice sheet modelling suggests that this mechanism predicts increases in ice mass loss across the AIS during the ACR in excess of 800 Gt/a, with an average of ~400 Gt/a, making a GMSL contribution of ~0.3 to 0.1 m per century^17^, importantly this rate almost doubles during the period defined by MWP-1A (Fig. 3E). Modelling of the following millennium implies a marked reduction in mass loss from all sectors of the AIS including the Weddell Sea (Fig. 3E), reflecting reduced Southern Ocean stratification and resumption of AABW formation post ACR, in agreement with our observations from the Patriot Hills BIA.

The coincidence between changes in AIS elevation from the Patriot Hills, enhanced iceberg flux^2^, atmospheric temperature trends^22^,^23^, and Southern Hemisphere mid-latitude westerly airflow^9^ through the LGT (Fig. 3B,C) implies a tight coupling between the ice-ocean-atmosphere system. Recent work using absolutely-dated tree ring chronologies has identified an abrupt increase in the inter-hemispheric radiocarbon gradient as a result of increased upwelling of ^14^CO_2_–depleted abyssal waters from 12.7 ka^12^, coincident with the maximum southerly extent of the Intertropical Convergence Zone (ITCZ) and strengthening Southern Hemisphere Westerlies (SHW)^9^. Our results are consistent with these findings, suggesting that weaker SHW during the ACR enhanced Southern Ocean stratification and maintained a positive ice-sheet-ocean feedback that drove substantial drawdown of the AIS (Fig. 4). This positive feedback appears to have only been disrupted by the re-expansion of the tropical belt and Hadley circulation during subsequent Northern Hemisphere cooling, and anti-phase southern warming after 12.7 ka (Fig. 3), suggesting AIS dynamics are highly sensitive to global atmospheric circulation.

The Patriot Hills preserves a record of significant AIS ice-sheet drawdown, mass loss and meltwater discharge during the ACR and across the LGT, contrasting markedly with previous interpretations of the configuration in the Weddell Sea sector of the AIS, which predict limited ice sheet drawdown since the local Last Glacial Maximum (LGM)^15^. Previous terrestrial reconstructions, based upon cosmogenic isotope analysis, predict a maximum thinning of ~480 m since the LGM, that occurred predominately during the mid-Holocene, suggesting that the WSE only made a minor contribution to GMSL rise since the LGM^25^. These estimates contrast with model-based reconstructions from far-field sites^16^, recent ice-sheet modelling studies^8^, reconstructions of IRD in the Scotia Sea^2^, and, crucially, our estimate of ~600 m of ice sheet surface elevation change across the ACR and MWP-1A (Fig. 3). Whilst we cannot define an upper altitudinal limit of the pre-ACR ice sheet across the WSE, the results reported here are inconsistent with estimates based upon terrestrial cosmogenic reconstructions^25^.

We suggest these contrasts may reflect two factors: firstly, there is a question over the effectiveness of terrestrial cosmogenic isotope studies to truly reflect the former elevation of the LGM ice-sheet surface in areas of cold based non-erosive polythermalice sheet settings^27^,^28^. Secondly, it is possible under a scenario of dynamic deglaciation during the LGT that rapid regional bedrock isostatic variations may have effectively masked rapid ice-sheet elevation changes that have occurred during deglaciation and subsequently during the Holocene (Supplementary Information). Therefore, terrestrial cosmogenic isotope reconstructions from mountains and nunataks across the WSE are only likely to robustly reconstruct ice-sheet surface elevation changes during Holocene deglaciation^25^,^29^,^30^. This is an issue which requires future detailed analysis, with multiple lines of evidence pointing towards a dynamic history of ice-sheet change across the WSE during the Holocene^29^,^30^,^31^, with significant implications for defining the pre-Holocene history of this sector of the AIS. Innovative reconstructions, such as that provided by the Patriot Hills BIA, are urgently required to define in detail dynamic Antarctic ice-climate feedbacks and better constrain the ice sheets contribution to global sea level rise during periods of rapid climate transition such as the LGT.

Supported by marine geological evidence of enhanced iceberg calving^2^, and independent ice-sheet and Earth system modelling experiments^17^, the Patriot Hills BIA provides the first direct terrestrial evidence that the Antarctic ice sheet was highly responsive to global ice-ocean-atmosphere feedbacks during the LGT^2^,^17^. Modelling suggests this pattern could be Antarctic wide, sustained by ice-ocean feedbacks amplified by the delivery of CDW onto the Antarctic Continental Shelf. The counterintuitive finding of sustained ice-sheet mass loss across this sector of the AIS during a period of atmospheric cooling suggests that Southern Ocean AIS feedbacks were likely modulated by global atmospheric teleconnections during a period of asynchronous hemispheric climate change. Defining the details of these atmosphere-ocean-ice feedbacks is crucial to reducing uncertainty in sea level projections^4^,^32^, and understanding the implications of observed high-latitude Southern Hemisphere environmental changes today^6^,^7^, which may conspire to amplify future Antarctic ice mass loss.

## Methods

### Description of the Patriot Hills BIA

The characteristics of the Patriot Hills BIA are rare in Antarctic terms, with Horseshoe Valley being a slow flowing (<5 m/a) compound glacier system situated within an over-deepened catchment, that coalesces with the Institute Ice Stream at the periphery of the WSE (Fig. 1), making it highly sensitive to grounding line changes across the WSE^33^; this contrasts with the relatively insensitive inland ice domes, the sites of traditional ice cores^23^ (Supplementary Information; Figure S8). In the lee of the Patriot Hills – a small mountain chain at the end of Horseshoe Valley – strong local katabatic winds descend into the valley from the polar plateau, ablating the ice sheet surface, drawing up ancient ice from depth within Horseshoe Valley, and forming the extensive (>800 m) Patriot Hills BIA^19^,^20^.

High-resolution analysis of the Patriot Hills BIA using ground-penetrating radar (GPR) demonstrates a remarkably consistent pattern of layering along the 800 m transect out from Patriot Hills with two distinct unconformities at 247 m (D1) and 360 m (D2) along the profile (Fig. 1). These unconformities are interpreted as periods of BIA formation within Horseshoe Valley^20^, occurring in the lee of mountains in the upper part of Horseshoe Valley during normal ice flow in the build-up to the LGM and at some point during the LGT. This interpretation is further supported by high-resolution ice-sheet modelling and GPR analysis, which concludes that there was no major regional flow direction change into Horseshoe Valley during the buildup of the AIS at the LGM^20^. Together, these lines of evidence confirm that the ice that accumulated between the unconformities at 247 m (D1) and 360 m (D2) is formed within the valley, thus providing a faithful record of environmental change in the catchment of Horseshoe Valley in response to broader changes across the WSE^19^,^20^,^21^.

### The chronology of the Patriot Hills BIA

Chronological control across the profile is provided by Antarctic-wide volcanic tephra horizons at 282 m (~17.8 ka), 279 m (18.2 ka) and 190 m (36.4 ka) (Supplementary Information), and a comprehensive suite of trace gas samples – carbon dioxide (CO_2_), methane (CH_4_) and nitrous oxides (N_2_O) – taken from depth (>3 m) along the BIA transect (Supplementary Information). The trace gases were extracted and measured at CSIRO’s Ocean and Atmosphere ICELAB facility in Melbourne, calibrated to internationally-recognised standards, and aligned to published values reported from EPICA Dome C^34^,^35^ (Supplementary Information), providing a conservative range of possible age solutions, which together with the absolute constraints provided by the tephra horizons, allows the development of a robust chronological framework (Supplementary Information) that can be tied directly to the isotopic profile through high-resolution GPR survey^19^,^20^. The integrity of the extracted air was further checked using sulfur hexafluoride (SF_6_) as an indicator of contamination by modern air. The average concentration of 8 samples analysed for SF_6_ was about 5% of modern day atmospheric concentrations and less than 2% for two of the samples selected to develop the chronology. The available constraints indicate the complete 800-m long Patriot Hills BIA transect spans ~50 to ~2.3 ka. Here we focus on the central section of the record, captured between the unconformities at 247 and 360 m, which, with multiple age ties together with the presence of the volcanic horizons dated to 17.8 ka and 18.2 ka, provides a robust chronology across the Patriot Hills BIA sequence of uninterrupted isochrons (Fig. 1). Further details are available in the Supplementary Information.

### Isotopic analysis

δD isotopic measurements at 1 m resolution were performed across the Patriot Hills BIA transect at the Australian Antarctic Division (AAD). These results were confirmed and augmented by δD and δ^18^O isotopic measurements at 5 m resolution at James Cook University (JCU), and the University of New South Wales (UNSW) ICELAB. At the AAD an on-line chromium reduction method on a EuroVector EuroPyrOH-HT system interfaced in continuous flow mode to an Isoprime isotope ratio mass spectrometer. Analytical precision is <0.5‰ and δD values are expressed relative to the Vienna Standard Mean Ocean Water (VSMOW) scale. To confirm δD values, particularly the rapid transitions across the periods defined by the LGT, δD and δ^18^O were measured independently at JCU using Diffusion Sampling - Cavity Ring-down Spectrometry (DS-CRDS). This system continuously converts liquid water into water vapour for real-time stable isotope analysis by laser spectroscopy (Picarro L2120-i, Sunnyvale, CA, USA). Each analytical run consisted of 12 standards interspersed with 44 unknown samples. Data processing was performed using a customised Excel™ template and included correction for between-sample memory, instrumental drift and normalisation to the VSMOW scale. Further details are available in the Supplementary Information. Finally, to ensure reproducibility a sub set of samples were rerun at UNSW ICELAB for δD and δ^18^O using a Las Gatos Research Liquid Water Isotope Analyzer 24d (International Atomic Energy WICO Lab ID. 16117). Reported overall analytical precision on long term ice core standards are <0.32‰ for δD, and <0.13‰ for δ^18^O values are expressed relative to the (VSMOW Scale).

## Additional Information

**How to cite this article**: Fogwill, C.J. *et al*. Antarctic ice sheet discharge driven by atmosphere-ocean feedbacks at the Last Glacial Termination. *Sci. Rep.* **7**, 39979; doi: 10.1038/srep39979 (2017).

**Publisher's note:** Springer Nature remains neutral with regard to jurisdictional claims in published maps and institutional affiliations.

## Supplementary Material

Supplementary Information
