# Supplementary material for: Antarctic ice sheet discharge driven by atmosphere-ocean feedbacks at the Last Glacial Termination
Source: Sci Rep. 2017 Jan 5;7:39979. doi: 10.1038/srep39979 (PMC5215443; doi:10.1038/srep39979)
Supplement: Supplementary Information [file srep39979-s1.pdf]

Antarctic ice sheet discharge driven by atmosphere-ocean feedbacks at the Last Glacial Termination

Authors: C.J. Fogwill*[1,2], C.S.M. Turney[1,2], N.R. Golledge[3,4], D. Etheridge[5], M. Rubino[5,6], D.P. Thornton[5], A. Baker[1], J. Woodward[7], K. Winter[7], T.D. van Ommen[8,9], A.D. Moy[8,9], M.A.J. Curran[8,9], S.M. Davies[10], M.E. Weber[11,12], M.I. Bird[13], N.C. Munksgaard[13,14], L. Menviel[1,2], C.M. Rootes[15], B. Ellis[16], H. Millman[2], J. Vohra [1,2], A. Rivera[17,18], A. Cooper[19]

Affiliations:

[1] PANGEA Research Centre, University of New South Wales, 2052, Australia

[2] Climate Change Research Centre, School of Biological Earth and Environmental Sciences, University of New South Wales, 2052, Australia

[3]Antarctic Research Centre, Victoria University of Wellington, Wellington 6140, New Zealand

[4]GNS Science, Avalon, Lower Hutt, New Zealand

[5]CSIRO Climate Science Centre, Oceans and Atmosphere, Aspendale, Victoria, 3195 Australia

[6]Dipartimento di Matematica e Fisica, Seconda Università di Napoli, viale Lincoln, 5-81100 Caserta, Italy

[7]Department of Geography, Faculty of Engineering and Environment, Northumbria

University, Newcastle upon Tyne, NE1 8ST, United Kingdom

[8]Australian Antarctic Division, 203 Channel Highway, Kingston, Tasmania 7050, Australia

[9]Antarctic Climate & Ecosystems Cooperative Research Centre, University of Tasmania, Private Bag 80, Hobart, Tasmania 7001, Australia

[10] Department of Geography, College of Science, Swansea University, Swansea, United Kingdom

[11]Department of Earth Sciences, University of Cambridge, Drummond Street, Cambridge, United Kingdom

[12]Steinmann Institute, University of Bonn, Poppelsdorfer Schloss, Bonn, Germany

[13]Centre for Tropical Environmental and Sustainability Science, College of Science and Engineering, James Cook University, Cairns, Australia

[14]Research Institute for the Environment and Livelihoods, Charles Darwin University, Australia

[15]Department of Geography, University of Sheffield, United Kingdom

[16]Research School of Earth Sciences, Australian National University, Canberra, Australia

[17]Glaciology and Climate Change Laboratory, Centro de Estudios Cientficos, Valdivia, Arturo Prat 514, Chile

[18]Department of Geography, University of Chile, Santiago, Chile

[19]Australian Centre for Ancient DNA, University of Adelaide, 5005, Australia

*Correspondence to: c.fogwill@unsw.edu.au

Supplementary Information:

Extended Methods

Figures S1 to S8:

Tables S1 to S5:

1. Description of the Patriot Hills BIA

The Patriot Hills BIA is rare in Antarctic terms, with Horseshoe Valley being a slow flowing (<5 m/a) compound glacier system situated within an over-deepened catchment, that coalesces with the Institute Ice Stream at the periphery of the WSE (main text Figure 1). In the lee of the Patriot Hills – a small mountain chain at the end of Horseshoe Valley – strong local katabatic winds descend into the valley from the polar plateau, ablating the ice sheet surface, drawing up ancient ice from depth within Horseshoe Valley, and forming the extensive Patriot Hills BIA which captures the 800 m wide Patriot Hills BIA[1-3].

High-resolution analysis of the Patriot Hills BIA using ground-penetrating radar (GPR) demonstrates a remarkably consistent pattern of layering along the 800 m transect out from Patriot Hills, interrupted by two distinct unconformities at 247 (D1) and 360 m (D2) along the profile (main text Figure 1). These unconformities are interpreted as periods of BIA formation within Horseshoe Valley[3], occurring in the lee of mountains in the upper part of Horseshoe Valley during normal ice flow in the build-up to the LGM and at some point during the LGT. The interpretation is further supported by high-resolution ice-sheet modelling and GPR analysis, which concludes that there was no major regional flow direction change into Horseshoe Valley during buildup of

the AIS at the LGM[3]. Together, these lines of evidence confirm that the ice that accumulated between the unconformities at 247 m and 360 m is local originating from Horseshoe Valley, thus providing a faithful recorder of environmental change in the catchment of Horseshoe Valley in response to broader changes across the WSE[2-4]. The end of Horseshoe Valley is buttressed by the Institute Ice Stream, with which local ice from the valley coalesces (Figure 1).

2. The chronology of the Patriot Hills BIA

A. Tephra extraction and analysis in the Patriot Hills BIA

The presence of visible tephras (volcanic ash marker horizons) from the Patriot Hills BIA allow us to generate a preliminary chronology for the Patriot Hills BIA. High concentrations of colourless and light brown glass particles were identified in 3 samples (282 m, 279 m and 190 m) investigated according to the presence of darker bands outcropping at the surface along the profile. Melted ice samples retrieved from below the surface with a Kovacs 9cm diameter ice corer were centrifuged at 2500 rpm, and the remaining particulate material was mounted on glass slides for electron microprobe analysis. The slides were ground using silica carbide paper and polished using 9, 6 and 1 μm diamond suspensions to produce fresh sections of the glass shards. Electron microprobe analysis was undertaken at the Tephrochronology Analytical Unit at the University of Edinburgh. Single-grain analyses of ten oxides were performed on a Cameca SX-100 instrument with 5 wavelength dispersive spectrometers. A 5 μm beam diameter was used and operating conditions followed those in [5]. Lipari Obsidian and BCR2G were analysed as secondary standards. A summary of the geochemical results is provided in Table S1.

Geochemical results reveal a bimodal composition for 282 m (Table S1) and single tightly-clustered trachytic populations for PH279 m and PH190 m (trachyte: PH282-a and rhyolite PH282-b) (Table S1). PH282-b, PH279 and PH190 show geochemical affinity to Mt Berlin products and to tephras identified in the Siple Dome core[6], Mt Moulton[7] and EPICA[8]. Similarity coefficient (SC) analysis[9] for PH190, highlight strong matches to a number of Siple Dome and Mt Moulton tephras ranging in age between 37 and 18 ka (Table S2). Several potential matches are revealed by SC values $\geq$0.95, with one sample, SDMA-5951c set 1 exhibiting a SC of 0.99 (Table S2A). Bivariate plots of samples with SC $\geq$ 0.97 for PH190 are shown in Figure S1. Although the data-sets overlap on several major elements we rule out SDMA-5635c on the basis of higher FeO_{tot} values, SDMA-9063b on higher SiO_2 values and slightly lower K_2O values and SDMA-5785c on lower SiO_2 values and slightly higher FeO_{tot} values (Figure S1). The data-set for SDMA-5951c set 1 exhibits a slightly wider range than PH190 but we believe that this is the most likely match. SDMA-5951c set 1 is dated to 36.4 ka yrs in the Siple Dome ice core[6].

The trachytic tephra PH279 most probably relates to volcanism in Marie Byrd Land[8]. Five tephras have SC values $\geq$ 0.95 with PH279 (Table S2B) and the compositional data for these are plotted on Figure S2. SDMA-9063b can be ruled out on the basis of higher SiO_2 values and EDC434.7 exhibits higher Al_2O_3 compositions. Although the remaining tephras overlap on many of the major elements, shards from PH279 are distinctly higher in Na_2O than SDMA-5554c and SDMA-5694. Although only summary statistics are available for WCM93-25, this is the only deposit to show partial overlap with PH279 on Al_2O_3 vs Na_2O and SiO_2 vs Na_2O plots (Figure S2). On this basis, we correlate PH279 with WCM-93-25 dated to 18.2 ka in the Mt Moulton record[7].

The rhyolitic component of the tephra at 282 m (PH282-a) is tightly clustered with average SiO_2 values of 74.6%, FeO_{tot} values of 2.1% and total alkali values of 9.7% (Table S1). We are unable to suggest a potential correlation for this tephra. In fact very few tephras of rhyolitic compositions are reported from the Antarctic ice-core records[6,8,10], and we suggest this tephra may originate from a volcanic source outside Antarctica. The trachytic population of PH282-b again shows considerable scatter and probably relates to volcanism in Marie Byrd Land[8], but we are unable to pinpoint a tephra of similar age and composition. As outlined we suggest that PH282-a and –b may relate to two separate eruptions in different volcanic systems. We suggest that PH282a and b most likely relates to widespread volcanic events dated to ~17.8 ka years and found in Byrd[11] and the WAIS divide[12] cores, which to date has not been geochemically typed but used as an Antarctic wide marker horizon by the ice coring community.

B. Extraction of trace gases from the Patriot Hills BIA.

Further chronological control across the profile is provided by a comprehensive suite of trace gas samples – carbon dioxide (CO_2), methane (CH_4) and nitrous oxides (N_2O) – taken from depth (>3 m) along the BIA transect. The trace gases were extracted and measured at CSIRO's Ocean and Atmosphere facility in Melbourne, and aligned to published values reported from EPICA Dome C, providing a range of possible age solutions, that together with the absolute constraint provided by the tephra horizons, allows the development of a robust chronological framework that can be tied directly to the isotopic profile through high-resolution GPR survey[2,3]. The available constraints indicate the complete 800 m long Patriot Hills BIA transect spans ~50 to ~2.3 ka (main text Figure 2). Trace gases were extracted from the ice, was undertaken at CSIRO Ice Lab using a dry extraction 'cheese grater' and cryogenic trapping technique[13], with minor alterations[14]. The

trapped air samples were analysed by gas chromatography (GC) and the trace gas concentrations are reported on the calibration scales maintained by CSIRO GASLAB[15].

The concept behind the dating routine is that CH_4 is the most reliable gas, as it is hard to be produced *in-situ* and it is generally not altered by post coring melting as much as CO_2 and N_2O. Therefore, we use "CH_4 only" to derive a first estimate of the age and age distribution. Then we use CO_2 and N_2O to better constrain the age distribution, only if they agree with the result provided by "CH_4 only". The criteria used to decide whether CO_2 and N_2O agree with the result from "CH_4 only" is that the ages estimated with the different gases differ either by less than 1000 years, or by less than 5000 years and the CO concentration is less than 120 ppb (high CO concentrations suggest possible *in-situ* production due to reaction of organics with hydrogen peroxide (H_2O_2)). Furthermore to assess possible modern atmospheric contamination we undertook analysis for the contaminant sulfur hexafluoride (SF_6) on a sample sub-set. The average concentration of eight samples analysed for SF6 was about 5% of modern day atmospheric concentrations, and less than 2% for the two samples selected to develop the chronology (with values ranging from 0.01-0.14 ppt). These thresholds have been chosen based on the minimum age difference that we aim to resolve and on the expectation of CO atmospheric values derived from past records[14].

The chronological framework for the Patriot Hills BIA profile was defined from the trace gases concentrations following nine key steps:

Step 1. <u>Assign a value and an uncertainty to each sample</u>: This is a critical step because the width of the distribution of gas concentration values determines the range of possible ages attributed to each sample.

The measured values have been corrected for systematic effects associated with the extraction procedure (named blank correction) according to the numbers in the headers of Table S3. Two different blank corrections have been used based on the period when the samples were extracted or analysed (2013 or 2015) due to changes in the extraction procedure. The value attributed to samples analysed in replicates is the average of multiple measurements. The uncertainty attributed to them is the difference between measured values. The uncertainty attributed to samples that were not measured in replicates is the average of the uncertainties shown in Table S3 (CH_4= 27 ppb, CO_2= 4 ppm; N_2O=2 ppb, see Table 4). Depending on the relative position and the slope of the spline fit to CH_4 measurements, the samples are assigned to a specific period (Glacial, Transition, Early or Late Holocene). These periods are converted into a start and an end of the possible gas age distribution based on the presence of tephra though the BIA profile, as shown in Table S4. While it was not possible to measure all samples in replicates, the agreement among age distributions suggested by multiple gases provide great confidence in our attributed ages

Step 2. Produce a Gaussian distribution for each gas species: With mean value equals the measured value and 1 sigma equals the uncertainty attributed (as shown in Table S4). This step allows us to attribute a probability to each possible range of values because the probability of obtaining a value in a certain concentration range is proportional to the area under the Gaussian distribution. The Gaussian distribution is divided in 100,000 intervals and covers a 3-sigma range (99.73 % of all possible values around the measured value).

Step 3. Fit the CH_4, CO_2 and N_2O records covering the last 120,000 years with a cubic spline: The gas records are a compilation of records from different ice cores. For CH_4, the EDC record has been taken. For CO_2, a compilation of records (mostly based on the EDC record[16]) has been

taken. For N_2O, the Talos Dome record has been taken[17]. The cubic spline has been produced by using the Matlab spline tool and choosing a time step of 10 years.

Step 4. Find the match between the Gaussian distribution and the spline fit for each species: For each gas age interval (x values on the spline fit plot), our dating routine checks whether there is a value of the Gaussian distribution that falls into the range of values (y values on the spline fit plot) defined by the start and end of the interval.

If a match is found, the probability for that value from the Gaussian distribution is attributed to the corresponding time interval. When more than one match is found for a specific gas age interval, the sum of the probabilities is taken. This procedure provides a way of projecting the probability from the Gaussian distribution onto the time axis (Figure S3). In other words we convert a "probability vs gas concentration" plot into a "probability vs gas age" plot for each gas species which quantifies the likelihood of a sample being x years old for each measured gas species. To represent a likelihood, the "probability vs gas age" plot (derived from each gas species) is normalised to a total area of 1.

Step 5: Find the intersection with the spline fit to the atmospheric records: Each measured value is projected to find the intersection with the atmospheric CH_4 spline fit. The most likely age is the intersecting value with the highest probability (mean value of the CH_4 age distribution), within the limit of the period that the sample falls in (Glacial, Deglacial, Early or Late Holocene, defined by the absolute chronological information define by available tephras). The same is performed with CO_2 and N_2O separately. If the most likely CO_2 age agrees with the most likely CH_4 age (according to the criteria described above), then a combined CH_4/CO_2 age is derived, based on the combined CH_4/CO_2 age distribution. If the most likely N_2O age agrees with the combined CH_4/CO_2 age, a combined $CH_4/CO_2/N_2O$ age is derived

Step 6. Force order in the age sequence: The sequence of samples is ordered chronologically from the oldest (PH-10) to the youngest (PH-800), meaning that e.g.: PH-270 must be younger than PH-200 and older than PH-290 and so on. This is used to constrain the most likely age attributed to each sample following the logical order based upon high-resolution GPR analysis[3] and the ages of the available tephra in the BIA profile.

Step 7: calculate the 1-sigma age range: Gas age ranges (68 % corresponding to 1 sigma) are calculated by integrating the area corresponding to 68 % of the total area of possible gas ages around the most likely age.

Step 8. Combine the likelihood from all species in one: The three gas species are assumed to provide independent evidence of likelihood of gas age and can be multiplied to get a combined likelihood (as when calculating the joint probability of independent events). We end up with a combined "probability vs gas age" plot. The area under the curve quantifies how likely it is that a sample is between x and x+Δx years old. Finally, the dating routine is run for all samples independently. This is summarised in Figure S4, the full sequence of samples, and can be seen against the EDC record of trace gases in Figure S5[18].

3. Constructing a geochronological framework across the profile

The final age model between the unconformities D1 and D2 can be seen in Table S5. The isotopic profile was corrected using the modelled bubble close off or Δage from the WDC core, which has the most tightly constrained Δage of any other regional ice core record[19]. The six well constrained tie-points that fall between the unconformities D1 and D2, lie across the Last Glacial Termination, and in conjunction with the age of well constrained volcanic horizons, recorded in other Antarctic ice cores (see section 2A) allow us to construct a robust chronology for the Patriot Hills BIA, and

particularly between unconformities D1 (247 m) and D2 (360m), which spans the period ~23 ka to 11 ka years (main text Figure 2 and Figures S5 and S6).

4. Isotopic analysis

δD isotopic measurements at 1m resolution were performed across the Patriot Hills BIA transect at the Australian Antarctic Division (AAD), James Cook University (JCU), and The University of New South Wales Ice Lab (UNSW ICELAB). At the AAD Isotope Laboratory using an on-line chromium reduction method on a EuroVector EuroPyrOH-HT system interfaced in continuous flow mode to an Isoprime isotope ratio mass spectrometer at the Australian Antarctic Division, in Hobart. Analytical precision is <0.5‰ and δD values are expressed relative to the Vienna Standard Mean Ocean Water 2 (VSMOW2).

To confirm δD values, particularly the rapid transitions across the period defined by the ACR, δD and $\delta^{18}O$ were measured independently at JCU using Diffusion Sampling - Cavity Ring-down Spectrometry (DS-CRDS[20]), to compare to other records and understand possible changes in precipitation sources as revealed from the Deuterium excess by regime shift analysis[21] (main text Figure 2 and Figure S6). This system continuously converts liquid water into water vapour for real-time stable isotope analysis by laser spectroscopy (Picarro L2120-i, Sunnyvale, CA, USA). Ice samples were thawed overnight and 5 mL transferred to auto-sampler vials covered with foil caps. An automated sampling system was used to pump each sample to the diffusion cell for isotopic measurement for 15 min. Each analytical run consisted of 12 standards interspersed with 44 unknown samples. Data processing was performed using a customised Excel™ template and

included correction for between-sample memory, instrumental drift and normalisation to the VSMOW scale.

The correction for memory effect was determined in a series of experiment with standard waters of known composition. It was found that 94 +/- 1% of the changes in $\delta^{18}O$ and δD values from the previous sample were recorded in the first replicate of a subsequent sample. This percentage remained constant regardless of the size or direction of change. Due to the volume requirement of the DS-CRDS instrument and small sample size (5 to10 mL) only one measurement could be made of most ice samples. Six vials of each of two working standards were analysed in each run: Casey Snow Melt ($\delta^{18}O$ = -18.02; δ^2H = -140.4) and Blue Ice ($\delta^{18}O$ = -39.14; δ^2H = -311.3).

The isotopic compositions of the working standards on the VSMOW scale were determined relative to IAEA standards VSMOW2, VSLAP2 and GISP by laser spectroscopy (Picarro L2120-i CRDS with injection/vaporisation attachment) and by IRMS (IAEA Stable Isotope Laboratory, Vienna). Replicate standard analyses yielded the following long term precision (1σ,10 runs): Casey Snow Melt: = 0.20 ‰, δD = 1.28 ‰, d-excess = 0.84 ‰ (n=43) and Blue Ice: $\delta^{18}O$ = 0.16 ‰, δ^2H = 1.06 ‰, δd-excess = 0.96 ‰ (n=46).

Finally, to ensure reproducibility a sub set of samples were rerun at UNSW ICELAB for δD and $\delta^{18}O$ using a Las Gatos Research Liquid Water Isotope Analyzer 24d (International Atomic Energy WICO Lab ID. 16117). Reported overall analytical precision is on long term ice core standards are <0.32‰ for δD, and <0.13 for $\delta^{18}O$ values are expressed relative to the Vienna Standard Mean Ocean Water 2 (VSMOW2).

5. Earth system and ice sheet modelling

Transient simulations of the last deglaciation was performed with the Earth System model LOVECLIM[22]. LOVECLIM comprises an ocean general circulation model and a thermodynamic-dynamic sea ice model, with an horizontal resolution of 3˚x3˚ and 20 vertical levels, coupled to a spectral T21quasi-geostrophic atmospheric model[23]. Starting from the background conditions of the Last Glacial Maximum, the model is forced with the time-varying evolution of solar insolation[24], ice sheet topography[25], high latitude albedo and atmospheric CO_2[26] for the period 21 ka to 10 ka. Here we compare simulations, one that incorporates a high southern latitude freshwater input (freshwater forcing (FWF)) at the time of the ACR[27], and one that does not (no freshwater forcing (NFW))[22] (Supplementary Fig. S7). As the two simulations differ only in the prescription (or not) of a Southern Ocean freshwater flux, they have been used to examine the ice-sheet response that arise solely as a consequence of this forcing[27]. In the first experiment (NFW) the millennial-scale variability of the last deglaciation is simulated by the addition of meltwater in the North Atlantic and/or the Southern Ocean. A weakening of the AMOC during the Younger Dryas is simulated by a 0.25 Sv freshwater flux applied in the Arctic Ocean (175˚W-95˚W, 67˚N-83˚N) between 13 and 12.2 ka Heinrich event 1 and the Younger Dryas are simulated by a weakening of the Atlantic Meridional Overturning Circulation obtained by freshwater input in the North Atlantic and Arctic, respectively. In the second experiment (FWF) the ACR is simulated by freshwater input in the Southern Ocean between 14.4 and 12.4 ka. The impacts of these experiments on a high-resolution ice sheet model is explored by Golledge *et al.*, 2014[27], and has been used to define grounding line migration, the sector wide mass flux from the AIS (main text Figure 3), and the effects on ocean temperatures across the Southern Ocean / Weddell Sea (main text Figure 4), and globally (Figure S6).

The changes in the geometry, dynamics and therefore mass of the AIS across the WSE predicted from independent whole Antarctic ice-sheet model experiments driven by the LOVECLIM FFW simulation outputs using the Parallel ice sheet model (PISM)[27] are consistent with both the timing and magnitude of ice-sheet surface elevation changes inferred from the Patriot Hills BIA (main text Figure 3). The periods of rapid surface elevation lowering (draw-down) across the region are characterised by the switching on and off of major ice streams across the Weddell Sea embayment (Figure S8), leading to enhanced mass loss and resultant sustained GMSL rise across the LGT focused on the ACR with a marked reduction in the following millennium (Younger Dryas Chronozone) (main text Figure 3).

This 'pulsed' or 'binge and purge' like response likely reflects strong sensitivity of the AIS to ocean feedback mechanisms, magnified by basal substrate / hydrology feedbacks that lead to ice streams switching on and off as the grounding line retreats across the variable sub-glacial topography of the WSE. During the 'on' phase ice streams exhibit high mass flux, leading to concomitant thinning over large areas of the ice sheet. This thinning reduces basal melt and substrate saturation, allowing the sub-glacial till to stiffen and eventually slowing ice flow, switching 'off' the ice stream. In their 'off' phase the ice thickens, increasing ice elevation regionally until enough basal melt is generated to induce saturation of the till, allowing thickening to start again. This behaviour is captured well in the high-resolution modelling experiments where ocean forcing and subglacial ice dynamic feedback mechanisms are included.

6. Discussion of dynamic behaviour of WAIS across the Weddell Sea

The dynamic nature of the surface profile changes suggested by our data and predicted by ice-sheet model simulations reflects sensitivity of the AIS to ocean forcing (main text Figure 3). This together with evolution of the basal substrate and hydrology, allows ice streams – the narrow conduits of fast flow which control the mass balance of the Antarctic ice sheet – to switch on and off. The ability to capture this evolving dynamic ice stream behaviour is critical, and enables detailed reconstruction of periods of rapid drawdown and flow direction changes to be achieved (Figure S8)[28]. This kind of behaviour is well documented across Antarctica and has been highlighted in paleo and contemporary ice-sheet studies[29], and is central to glaciological theory[30]. When the ice streams are in their off state, regionally the ice sheet catchment thickens substantially, until plastic till failure occurs and effectively switches them back on, as recorded on the Siple Coast ice streams today[29].

Prior to discussing the implications of the results it is critical to address potential factors that impact our interpretation. Two important questions that must be addressed. Firstly that of potential ice advection from outside Horseshoe Valley. Secondly the contrast between the reconstruction presented here, and by high-resolution modelling and those derived from previous terrestrial reconstructions of the LGM WAIS configuration in the Weddell Sea based upon terrestrial cosmogenic isotope analysis and marine reconstructions[31].

The question over ice advection from outside Horseshoe Valley is crucial, as it impacts the interpretation of the elevation changes recorded in the ice exposed at the Patriot Hills BIA, and therefore any WAIS ice dynamic changes predicted. Key to understanding this are insights into regional ice sheet dynamics derived from the interpretation of airborne radar, specifically radio

echo sounding (RES), which investigate variations in the internal layering characteristics of ice across the WAIS with depth. Analysis and detailed interrogation of available RES predicts that the region of the Ellsworth Mountains, and particularly in the area of Horse Shoe Valley is an area of 'unusually' low ice flow, with predicted velocities today of <5 m a^{-1}. These low velocities are a direct result of flow near the substantial sub-glacial topographic obstacles that the Ellsworth Mountains represent[32]. This is supported by other geophysical structural geological interpretations which demonstrated that the Ellsworth Trough, abutting the Ellsworth-Whitmore Mountain Block, preferentially drains ice from the WAIS interior down into the Institute Ice Stream[33], effectively isolating Horseshoe Valley, today, during the Holocene[32], and into the LGM[3]. Thus making Horseshoe Valley ideal for preserving a long-paleoclimate record from the BIA, but unfortunately not ideal for attempting to reconstruct pre-Holocene regional ice sheet dynamic history from cosmogenic isotope analysis[31,34,35].

This point leads directly to the second important question, that of why the elevations predicted from our interpretation of the isotopes captured in the record contrast markedly with previous interpretations of the maximum LGM configuration in the Weddell Sea which predict limited ice sheet drawdown since the local LGM[31,34,35]. Past terrestrial reconstructions predict maximum thickening of ~480 m at the LGM based upon cosmogenic isotope analysis, suggesting the Weddell Sea has only made a minor contribution to GMSL rise since the LGM, whilst modelled reconstructions suggesting that the Weddell Sea contributed between 1.4 to 2 m to postglacial sea-level rise[36]. These estimates contrast markedly with model-based reconstructions from far-field sites[37], recent ice sheet modelling studies[27], reconstructions of IRD in the Scotia Sea[38], and our estimates of ~600 m of surface elevation change across the ACR and MWP-1A. We suggest these

contrasts reflect two important factors: firstly, there is a growing body of evidence that surface exposure dates do not necessarily reflect the true former elevation of the LGM ice-sheet surface in areas of cold based non-erosive ice[35], which has been demonstrated in many former ice sheet settings[39]. Secondly, as discussed, it is possible that due to the dynamic nature of Antarctic ice sheet, with the switching on and off of the ice streams during retreat and post LGM ice sheet evolution, dynamic changes may effectively mask rapid ice-sheet elevation changes that may have occurred during deglaciation. Therefore, terrestrial cosmogenic isotope reconstructions from Antarctic nunataks are likely to only robustly reconstruct the final stages of dynamic Holocene deglaciation[28].

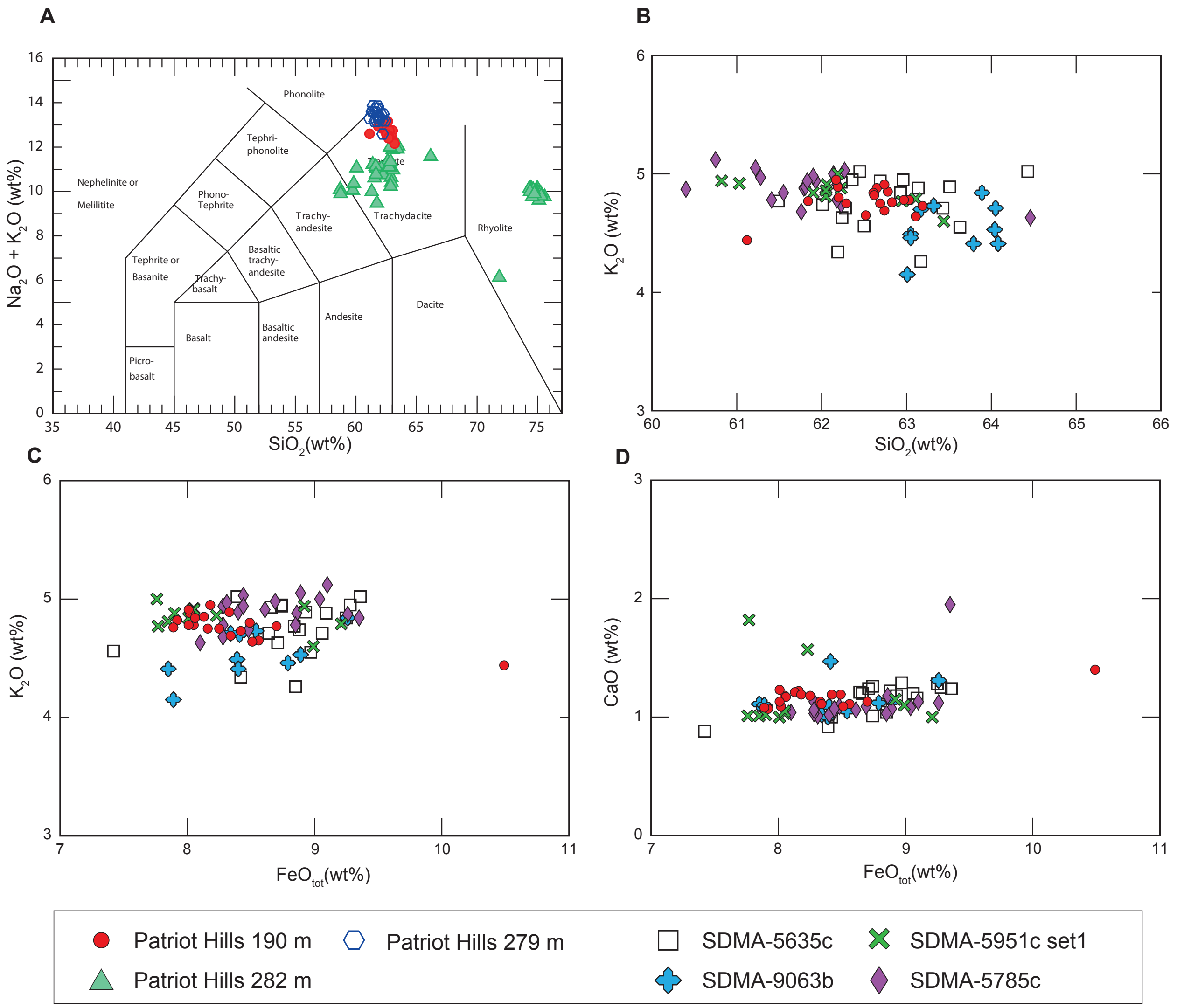

Figure S1. Examples of major element results for Patriot Hills tephra samples at 190 and 282 and 279m. Data are normalised and expressed as weight %. Siple Dome tephra data are from[6], and for clarity only those with similarity coefficient values ≥ 0.97 with PH190 are shown (see Table S2).

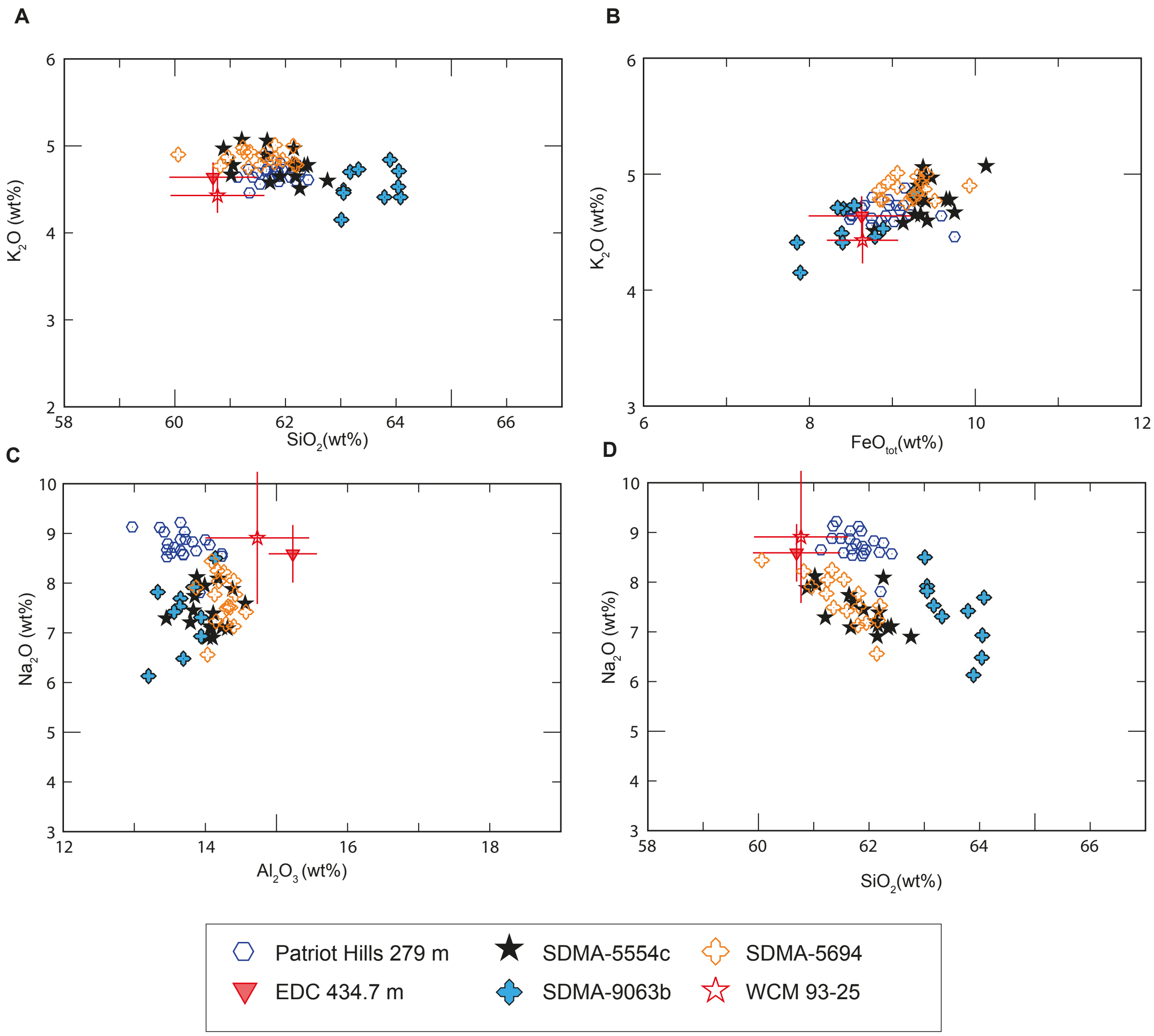

Figure S2. Examples of major element results for Patriot Hills tephra samples at 279 m. Data are normalised and expressed as weight %. Siple Dome tephra are from[6], EPICA Dome C are from[8] and WCM93-25 is from[7]. Only those with similarity coefficient values ≥0.95 are shown (see Table 2B).

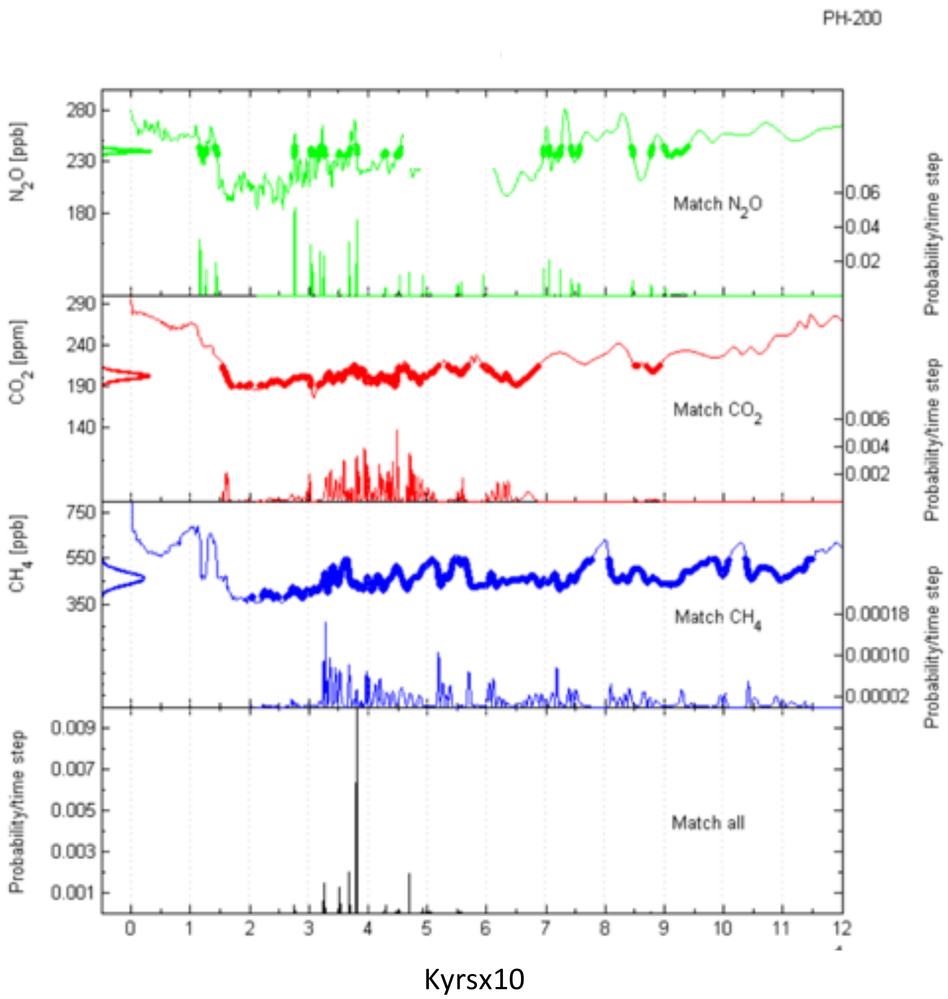

Figure S3. Plot of the normalised "probability vs gas age" distribution for PH-200. x-axis Kyrs*10 Before Present (present is 1950 AD). Upper y-axes of each box are concentrations whereas lower y-axes of each box are probabilities of a) N_2O, b) CO_2 and c) CH_4. Plot d) gives the combined probability given by the products of all probabilities in plot a), b) and c).

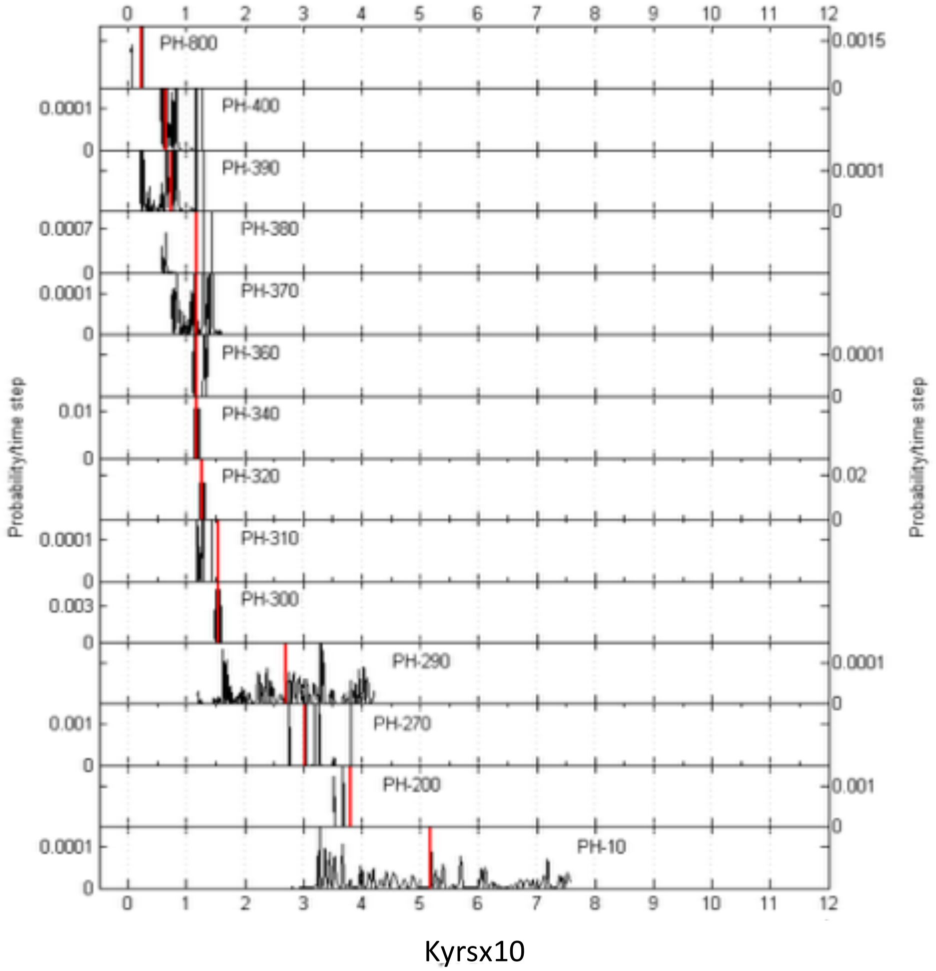

Figure S4. Probability plot of gas age and gas age range of all PH samples in x-axis Kyrs*10 Before Present (present is 1950 AD). The precision and accuracy of our dating routine depends on the slope of change of trace gas concentrations. For periods where the change is steep (e.g.: the transition), the precision is high, as demonstrated by the narrow age distributions of samples PH-300 to PH-360 in Figure 2.

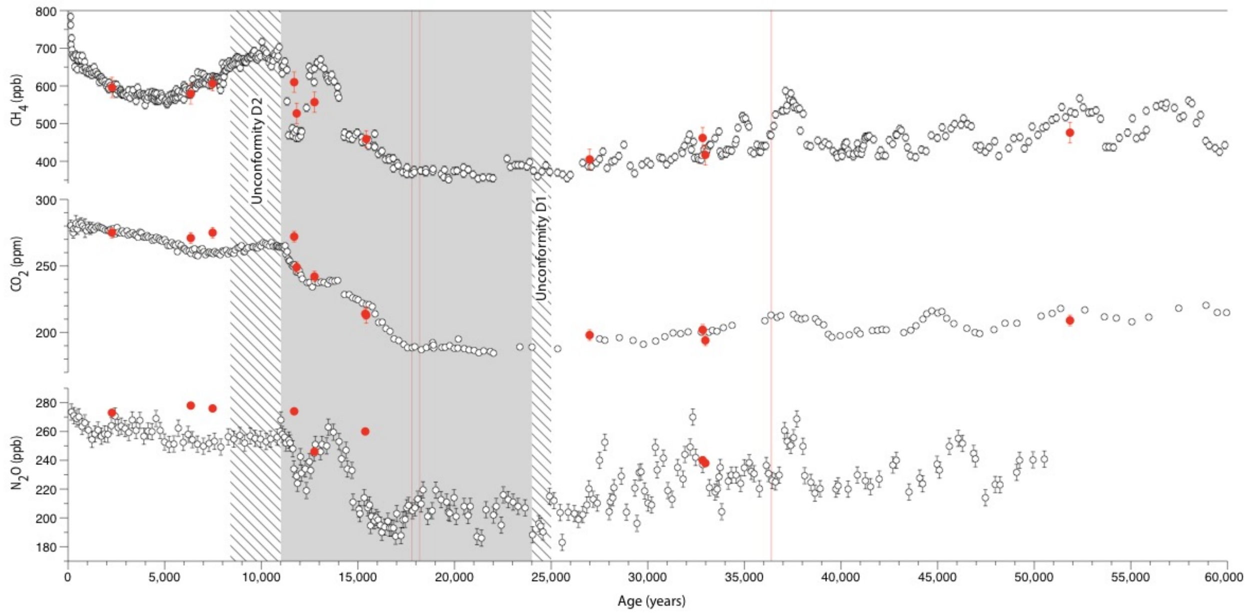

Figure S5. CH_4 (upper panel), CO_2 (middle panel) and N_2O (lower panel) gas concentrations from ice extracted from the Patriot Hills (red circles) profile plotted against concentrations EDC (EDC1 timescale; black circles)[16,17,18,19]. Vertical red lines mark the timing of the geochemically identified tephra across the profile.

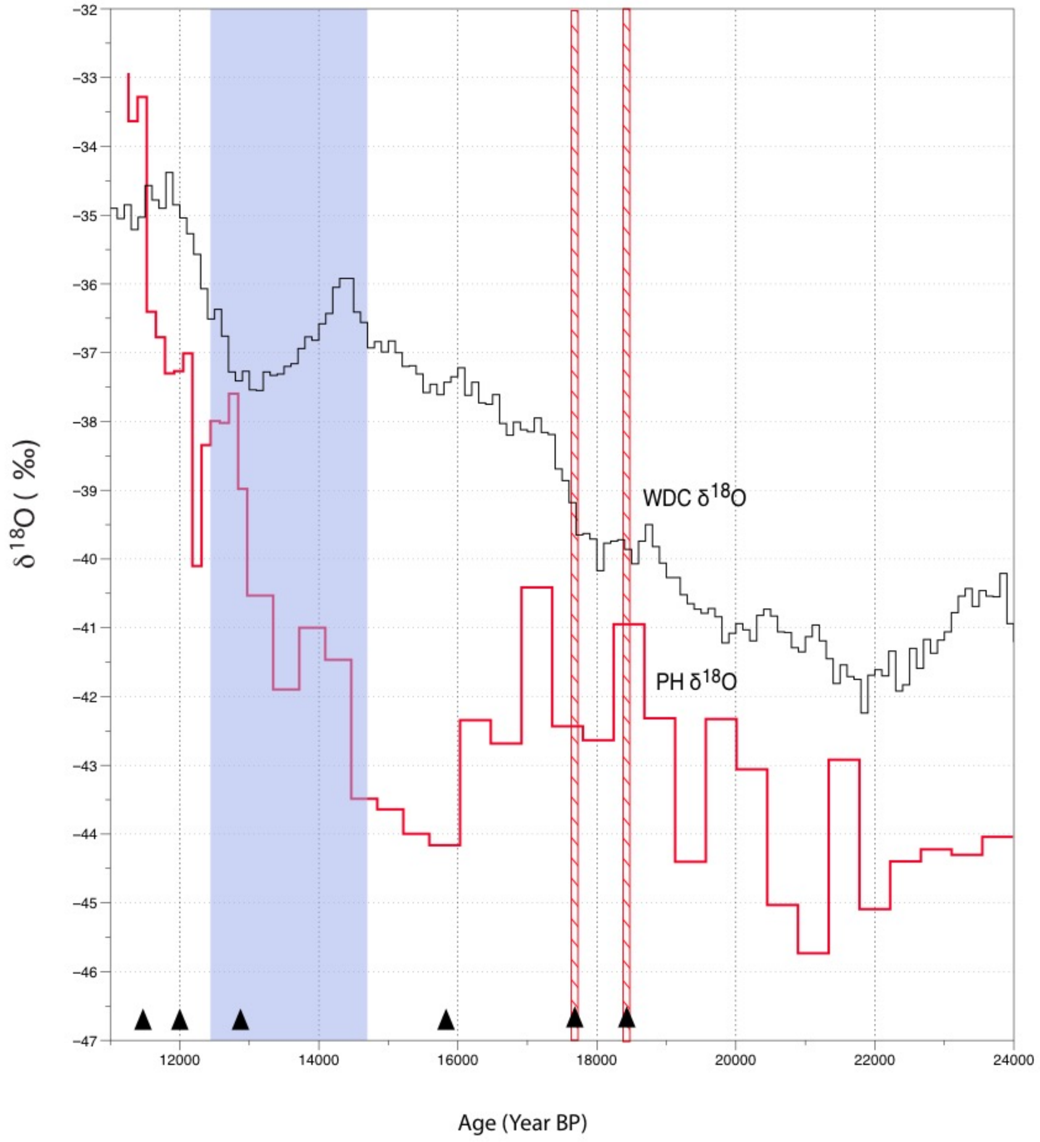

Figure S6. Detailed inter-comparison of the WAIS Divide Core $\delta^{18}O$ isotopic record[12] (in black to WDC 2014 Chronology) with the 5 m resolved $\delta^{18}O$ profile from the Patriot Hills BIA (in red) profiles defined between 11,000 and 24,000 years (between unconformities D1 and D2). The light blue boxes define the period defined as the ACR[40]. The black triangles define the age ties across the Patriot Hills BIA.

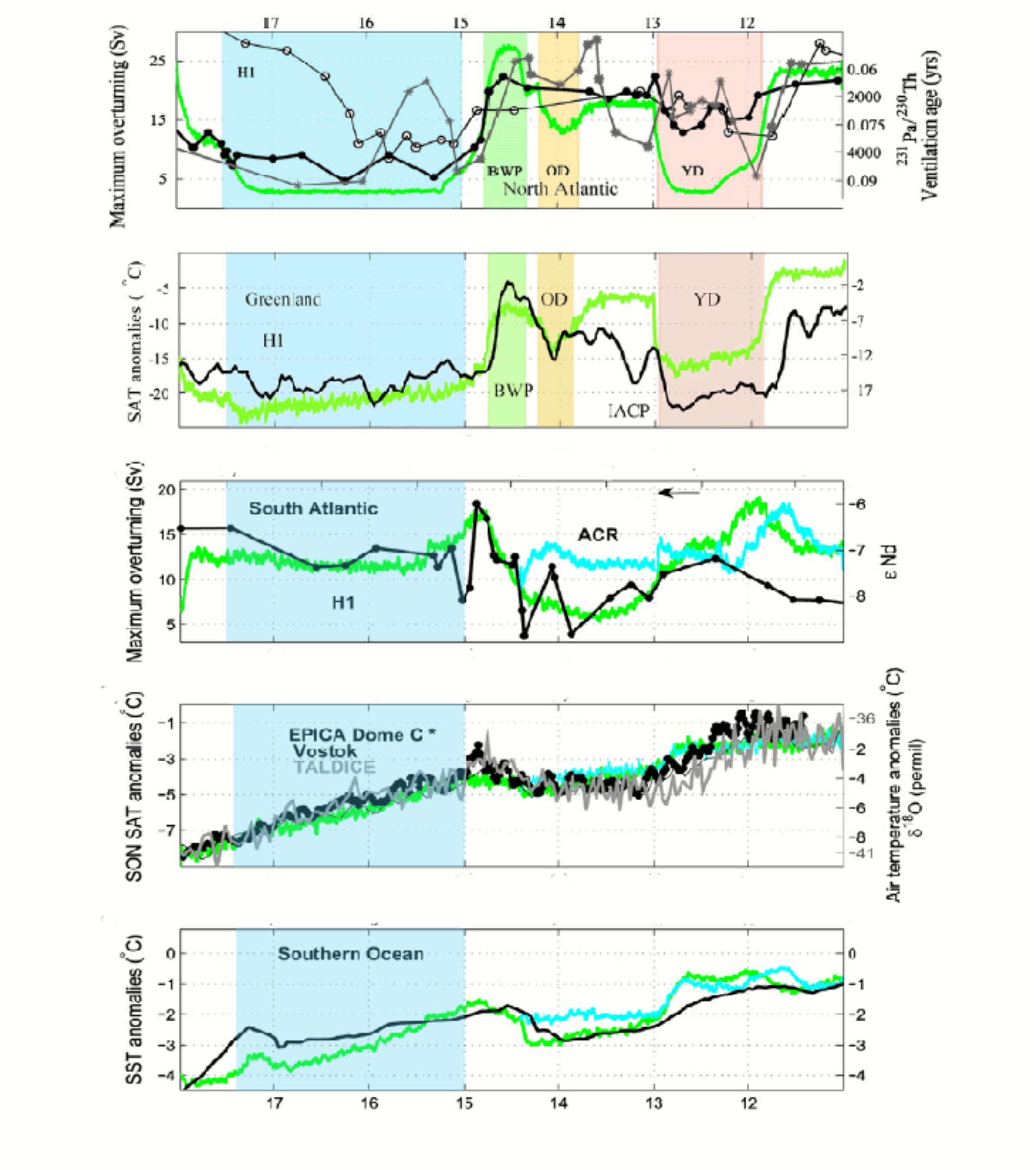

Figure S7. Transient deglaciation experiments performed with LOVECLIM compared to proxy records, where proxy records are in black / grey (Menviel *et al.*,[22] and references therein), LOVECLIM NFW experiment (no fresh water forcing in Southern Ocean) in blue, and LOVECLIM FWF (with 0.15 Sv in Ross and Weddell seas) in green. From top down: North Atlantic Overturning (Sv) (compared to $^{231}Pa/^{230}Th$ data (black) from core OCE326-GGC5 (filled circles) and from core SU81-18 (empty circles) as well as ventilation age data (gray) from cores RAPID 10-1P, 15-4P and 17-5P. H1 stands for Heinrich event 1,

BWP for Bølling Warm Period, OD for Older Dryas and YD for Younger Dryas), North Atlantic Sea Surface Temperatures (SST) (˚C), South Atlantic Overturning (Sv), Surface air temperature anomalies (˚C), Southern Ocean Sea Surface Temperatures (SST) (˚C).

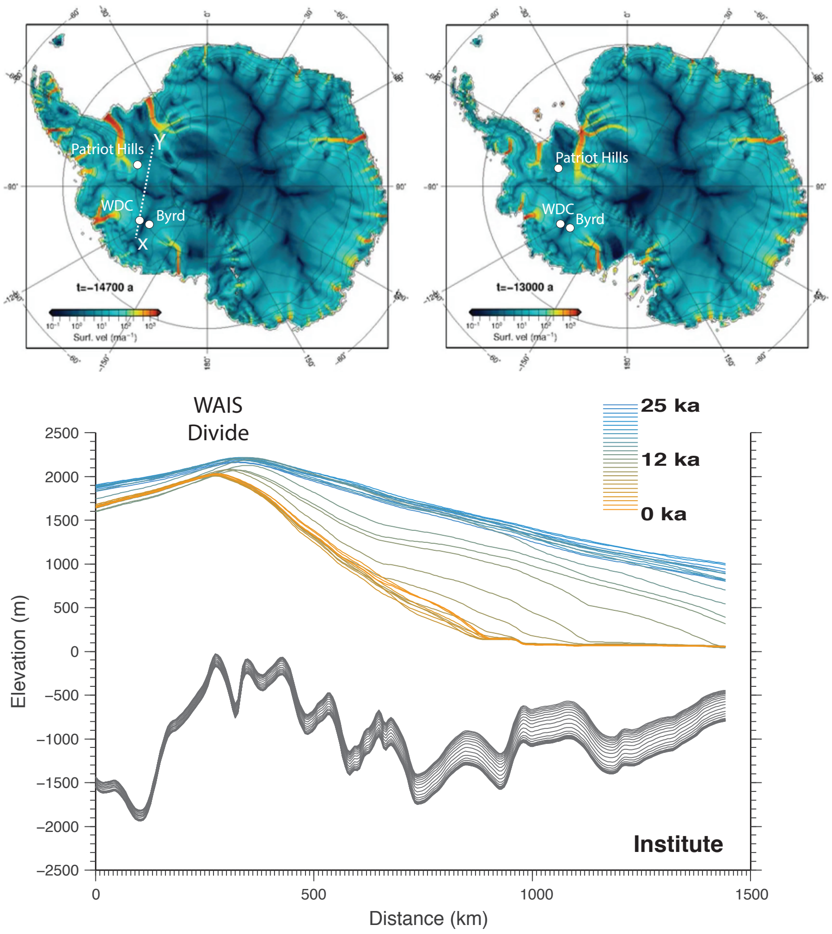

Figure S8. Upper panel: Predicted surface velocity changes at the start (14.7 ka) and the end (13 ka) of the ACR derived from independent ice sheet model experiment (using PISM v0.6), with fresh water forcing in the Southern Ocean[27], demonstrating the migration and evolution of ice streams across the WSE during this period of AIS mass loss. Lower panel: Transect along the Institute ice stream showing the evolution of the ice stream and the bedrock through time from the LGM to present day as predicted from the PISM model experiments,

demonstrating the limited altitudinal change predicted at the WAIS Divide core (WDC) site (see upper panel for approximate X-Y line of cross section).

Tables

Table 1. *Summary geochemistry for Patriot Hills tephra deposits. Data (weight %) are normalized and presented as mean and 1 standard deviation. Original totals are also shown. n = number of tephra shards analysed from each sample. Geochemical classification follows* **Le Maitre[41].** *PH282 and 190 were analysed on 7/11/15 and PH279 on 29/2/16 and secondary standard data for those two different analytical periods are shown. Recommended values for the Lipari are from* Kuehn et al.,[42] and the BCR2G from Wilson[43]

Sample	n	SiO_2	TiO_2	Al_2O_3	FeO_{tot}	MnO	MgO	CaO	Na_2O	K_2O	P_2O_5	Total	Classification
PH282-a	18	74.66	0.12	13.00	2.07	0.05	0.04	0.34	5.46	4.25	0.01	98.45	Rhyolite
st.dev.		0.78	0.12	0.41	0.32	0.02	0.17	0.63	0.27	0.65	0.03	0.72	
PH282-b	27	62.01	0.66	16.92	5.80	0.18	0.46	2.86	6.18	4.71	0.23	99.32	Trachyte
st.dev.		1.59	0.30	1.56	1.72	0.05	0.27	0.95	0.50	0.91	0.23	0.47	
PH279	22	61.78	0.53	13.69	8.90	0.37	0.04	1.22	8.75	4.67	0.05	99.62	Trachyte
st.dev		0.33	0.02	0.30	0.34	0.01	0.02	0.06	0.29	0.09	0.01	0.62	
PH190	19	62.54	0.48	14.34	8.34	0.35	0.06	1.16	7.89	4.77	0.06	98.73	Trachyte
st.dev		0.49	0.02	0.34	0.57	0.03	0.02	0.08	0.24	0.12	0.01	1.12	
Secondary standard data													
Lipari (7/11/15)	7	74.37	0.08	12.89	1.49	0.07	0.04	0.78	3.90	5.20	0.01	98.82	
st.dev		0.52	0.00	0.17	0.12	0.01	0.02	0.04	0.10	0.08	0.01	0.78	
Lipari (29/2/16)	*8*	74.23	0.08	13.03	1.57	0.07	0.05	0.74	4.13	5.21	0.01	99.11	
stdev		0.66	0.00	0.12	0.07	0.01	0.01	0.01	0.13	0.1	0.1	0.01	
Recommended range		*73.14-75.06*	*0.05-0.10*	*12.77-13.45*	*1.49-1.61*	*0.040-0.1*	*0.02-0.06*	*0.68-0.79*	*3.78-4.34*	*4.87-5.39*	*0.00-0.02*		
BCR2G (7/11/15)	8	54.73	2.29	13.19	12.57	0.20	3.54	7.11	3.07	1.81	0.34	98.84	
st.dev		0.64	0.01	0.17	0.25	0.01	0.07	0.10	0.18	0.03	0.01	0.52	
BCR2G (29/2/16)	13	54.23	2.28	13.39	12.47	0.2	3.63	7.24	3.15	1.82	0.33	98.74	
stdev		0.43	0.01	0.16	0.26	0.01	0.07	0.15	0.3	0.05	0.03	0.47	
Recommended		*54.1*	*2.26*	*13.5*	*12.42*	*n.d.*	*3.59*	*7.12*	*3.16*	*1.79*	*0.35*		
st.dev		*0.8*	*0.05*	*0.2*	*0.2*		*0.05*	*0.11*	*0.11*	*0.05*	*0.02*		

Table S2. Table 2. A) Similarity coefficient analysis of Patriot Hills 190 m and Siple Dome tephras ranging in age between 37 and 18 ka[34]. B) Similarity coefficient analysis of Patriot Hills 279 m, Siple Dome, EDC and Mount Moulton tephras younger than 37 ka[6]. Only the most likely tephra matches are shown (>0.95). Similarity coefficient follows that outlined in[9]. For clarity, only the values shown in bold are plotted in Figures 2 and 3.

A **B**

PH190m	SC	PH279m	SC
SDMA-5521c	0.95	EDC434.7	0.96
SDMA-5554c	0.96	SDMA-5554c	0.95
SDMA-5620c	0.96	SDMA 9063	0.95
SDMA-5630c	0.95	SDMA 5694	0.95
SDMA-5635c	0.97	**WCM93-25**	**0.95**
SDMA-9063b	0.97		
SDMA-5694	0.96		
SDMA-5785c	0.97		
SDMA-9065b	0.95		
SDMA-5824c set 1	0.96		
**SDMA-5951c set 1**	**0.99**		

Table S3: Corrected measured values of trace gases from CSIRO Ice Lab. The core number represents the distance (in meters) of each sample along the transect. Measured values are given for each gas species (methane, CH_4, carbon dioxide, CO_2, and nitrous oxide, N_2O) and for each core number. Some core numbers miss the corresponding N_2O value because the amount of air available was enough to analyse only CH_4 and CO_2. Uncertainties are given only for samples measured in replicates. The age constraint column provides the geological epoch each sample is assumed to belong to based on consideration of the CH_4 spline trend. Carbon monoxide (CO) is used as a diagnostic tool for contamination / *in-situ* production. One core number misses the corresponding CO value because of technical issues with the GC measuring CO concentrations.

Core number	CH4 [ppb]	Δ [ppb]	CO2 [ppm]	Δ [ppm]	N2O [ppb]	Δ [ppb]	Age range start	Age range end	CO [ppb]
10	476	27	209	4			120000	19500	154
200	462	28	202	4	240	2	120000	19500	92
270	417	27	194	4	238	2	120000	19500	95
290	405	27	198	4			120000	11000	173
300	459	22	213	6			19500	11000	
310	528	27	214	4	260	2	19500	11000	179
320	557	27	242	4	246	2	19500	11000	169
340	527	27	249	4			19500	11000	60
360	610	27	272	4	274	2	19500	11000	64
370	602	57	271	7	275	1	19500	5500	53
380	582	10	270	1	279	2	19500	5500	76
390	606	19	275	4	276	2	19500	5500	68
400	579	27	271	4	278	2	19500	5500	80
800	596	27	275	4	273	2	11700	10	75

Table S4. Complete dataset used to develop the trace gases based chronological ties.

Core number	CH4 (average) - 4/22 [ppb]	Δ [ppb]	CO2 (average) - 0.7/1.6 [ppm]	Δ [ppm]	N2O (average) - 1.9 [ppb]	Δ [ppb]	Age constraint based on CH4 spline slope	CO (average) - 10 [ppb]
10	476		209				Glacial	154
200	462	28	202	4	240		Glacial	92
270	417		194		238		Glacial	95
290	405		198				Glacial/Transition	173
300	459	22	213	6			Transition	
310	528		214		260		Transition	179
320	557		242		246		Transition	169
340	527		249				Transition	60
360	610		272		274		Transition	64
370	602	57	271	7	275	1	Transition/Early Holocene	53
380	582	10	270	1	279	2	Transition/Early Holocene	76
390	606	19	275		276		Transition/Early Holocene	68
400	579		271		278		Transition/Early Holocene	80
800	596		275		273		Late Holocene	75

Table S5. Chronological tie points with minimum uncertainty accepted across the Patriot Hills profile used to develop age model across profile in years BP. The chronological control points between the unconformities D1 at 247 m and D2 360 m are highlighted in green.

Distance along profile (depth / dip corrected)	Δage (years) WDC	upper age (years)	lower age (years)	Attributed corrected age	Type of constraint trace gas, tephra or volcanic horizon
10 (10)	320	75,660	28.040	52,170	Trace gas
190 (190)	N/A	N/A	N/A	36,400	Tephra
200 (200)	400	40,970	35,190	33,290	Trace gas
279 (279)	N/A	N/A	N/A	18,200	Tephra
282 (282)	N/A	N/A	N/A	17,800	Volcanic horizon
300 (297)	250	15,580	15,280	15,430	Trace gas
320 (318)	220	12,770	12,730	12,750	Trace gas
340 (339)	220	11,880	11,780	11,830	Trace gas
360 (357)	220	13,790	11,000	11,700	Trace gas
390 (384)	150	13,010	1,950	7,480	Trace gas
400 (394)	150	12,940	5,500	6,350	Trace gas
800 (792)	150	4,230	550	2,390	Trace gas

References

1 Fogwill, C. J., Hein, A., Bentley, M. J. & Sugden, D. E. Do blue-ice moraines in the Heritage Range show the West Antarctic Ice Sheet survived the last interglacial? *Palaeogeography, Palaeoclimatology, Palaeoecology* **335–336** (2012).

2 Turney, C. S. M. *et al.* Late Pleistocene and early Holocene change in the Weddell Sea: a new climate record from the Patriot Hills, Ellsworth Mountains, West Antarctica. *Journal of Quaternary Science* **28**, 697-704 (2013).

3 Winter, K. *et al.* Assessing the continuity of the blue ice climate record at Patriot Hills, Horseshoe Valley, West Antarctica. *Geophys. Res. Lett.* **In press** (2016).

4 Hein, A. S. *et al.* Evidence for the stability of the West Antarctic Ice Sheet divide for 1.4 million years. *Nature Communciations* **7** (2016).

5 Haywood, C.L., *et al*., High spatial resolution electron probe microanalysis of tephras and melt inclusions without beam-induced chemical modifications. *The Holocene* **22**, 119-125.

6 Dunbar, N. & Kurbatov, A. Tephrochronology of the Siple Dome ice core, West Antarctica: correlations and sources. *Quaternary Science Reviews* **30**, 1602-1614 (2011).

7 Wilch. T.I., McIntosh, W. C. & Dunbar, N. W. Late Quaternary volcanic activity in Marie Byrd Land: Potential 40Ar/39Ar-dated time horizons in West Antarctic ice and marine cores. *GSA Bulletin* **111**, 1563-1580 (1999).

8 Narcisi, B., Petit, J., Delmonte, B. & et al. Characteristics and sources of tephra layers in the EPICA-Dome C ice record (East Antarctica): Implications for past atmospheric circulation and ice core stratigraphic correlations. *Earth and Planetary Science Letters*, 253-265 (2005).

9 Borchardt, G., Aruscavage, P. & Millard, H. Correlation of the Bishop ash, a Pleistocene marker bed, using instrumental neutron activation analysis. *Journal of Sedimentary Petrology* **42**, 301-306 (1972).

10 Narcisi, B., Petit, J., Delmonte, B. & al., e. A 16,000-yr tephra framework for the Antarctic ice sheet: a contribution from the new Talos Dome core. *Quaternary Science Reviews* **49**, 52-63 (2012).

11 Hammer, C. U., Clausen, H. B. & Langway, C. C. 50,000 years of global volcanism. *Climatic Change* **35** (1997).

12 WAIS Divide Members. Onset of deglacial warming in West Antarctica driven by local orbital forcing. *Nature* **500**, 440-444 (2013).

13 Etheridge, D. M. *et al.* Natural and anthropogenic changes in atmospheric CO2 over the last 1000 years from air in Antarctic ice and firn. *Journal of Geophysical Research* **101**, 4115-4128 (1996).

14 Rubino, M. *et al.* A revised 1000 year atmospheric δ13C-CO2 record from Law Dome and South Pole, Antarctica. *Journal of Geophysical Research: Atmospheres* **118**, 8482-8499, (2013).

15 Francey, R. J. *et al.* The CSIRO (Australia) measurement of greenhouse gases in the global atmosphere, report of the 11th WMO/IAEA Meeting of Experts on Carbon Dioxide Concentration and Related Tracer Measurement Techniques, Tokyo, Japan, September 2001, S. Toru and S. Kazuto (editors), World Meteorological Organization Global Atmosphere Watch, 97-111. (2003).

16 Lüthi, D. *et al.* High-resolution carbon dioxide concentration record 650,000-800,000 years before present. *Nature* **453**, 379-382, (2008).

17 Schilt, A. *et al.* Atmospheric nitrous oxide during the last 140,000 years. *Earth and Planetary Science Letters* **300**, 33-43 (2010).

18 Monnin, E. *et al.* Evidence for substantial accumulation rate variability in Antarctica during the Holocene, through synchronization of CO2 in the Taylor Dome, Dome C and DML ice cores. *Earth and Planetary Science Letters,* **224**, 45-54 (2004).

19 Buizert, C. *et al.* The WAIS Divide deep ice core WD2014 chronology; Part 1: Methane synchronization (68–31 ka BP) and the gas age–ice age difference. *Clim. Past* **11**, 153-173, (2015).

20 Munksgaard, N.C., Wurster, C. M. & M.I. Bird. Continuous analysis of d18O and dD values of water by diffusion sampling cavity ring-down spectrometry: a novel sampling device for unattended field monitoring of precipitation, ground and surface waters. *Rapid Communications in Mass Spectrometry* **25**, 3706-3712 (2011).

21 Rodionov, S. N. A sequential algorithm for testing climate regime shifts. *Geophys. Res. Lett.* **31** (2004).

22 Menviel, L., A. Timmermann, O. Elison Timm & Mouchet, A. Deconstructing the Last Glacial Termination: the role of millennial and orbital-scale forcings. *Quaternary Science Reviews* **30**, 1155-1172 (2011).

23 Goosse, H. *et al.* Reconstructing surface temperature changes over the past 600 years using climate model simulations with data assimilation. *J. Geophys. Res* **115** (2010).

24 Berger, A. L. Long-Term Variations of Daily Insolation and Quaternary Climatic Changes. *Journal of the Atmospheric Sciences* **35**, 2362-2367 (1978).

25 W.R. Peltier. Ice Age Paleotopography. *Science* **265**, 195-201 (1994).

26 Monnin, E. *et al.* Atmospheric CO2 Concentrations over the Last Glacial Termination. *Science* **291**, 112-114 (2001).

27 Golledge, N. R. *et al.* Antarctic contribution to meltwater pulse 1A from reduced Southern Ocean overturning. *Nat Commun* **5**, doi:10.1038/ncomms6107 (2014).

28 Fogwill, C. J. *et al.* Drivers of abrupt Holocene shifts in West Antarctic ice stream direction determined from combined ice sheet modelling and geologic signatures. *Antarct. Sci.* **26**, 674-686, (2014).

29 Hulbe, C. & Fahnestock, M. Century-scale discharge stagnation and reactivation of the Ross ice streams, West Antarctica. *J. Geophys. Res. Earth Surf.* **112 (F3)**, F03S27 (2007).

30 Schoof, C. A Variational Approach to Ice Stream Flow. *Journal of Fluid Mechanics* **272 556**, 227-251 (2006).

31 Bentley, M. J. *et al.* Deglacial history of the West Antarctic Ice Sheet in the Weddell Sea embayment: Constraints on past ice volume change. *Geology* **38**, 411-414 (2010).

32 Bingham, R. G. *et al.* Ice-flow structure and ice dynamic changes in the Weddell Sea sector of West Antarctica from radar-imaged internal layering. *Journal of Geophysical Research: Earth Surface* **120**, 655-670. (2015).

33 Jordan, T. A. *et al.* Inland extent of the Weddell Sea Rift imaged by new aerogeophysical data. *Tectonophysics* **585**, 137-160, (2013).

34 Bentley, M. J. *et al.* Deglacial history of the West Antarctic Ice Sheet in the Weddell Sea embayment: Constraints on past ice volume change: REPLY. *Geology* **39**, e240, doi:10.1130/g32140y.1 (2011).

35 Clark, P. U. Deglacial history of the West Antarctic Ice Sheet in the Weddell Sea embayment: Constraints on past ice volume change: Comment:. *Geology* **39** (2011).

36 Le Brocq, A. M. *et al.* Reconstructing the Last Glacial Maximum ice sheet in the Weddell Sea embayment, Antarctica, using numerical modelling constrained by field evidence. *Quaternary Science Reviews* **30(19-20)** (2011).

37 Clark, P. U., Mitrovica, J. X., Milne, G. A. & Tamisiea, M. Sea level fingerprinting as a direct test for the source of global meltwater pulse IA,. *Science* **295**, 2438-2441 (2002).

38 Weber, M. E. *et al.* Millennial-scale variability in Antarctic ice-sheet discharge during the last deglaciation. *Nature* **510**, 134-138, doi:10.1038/nature13397 (2014).

39 Fabel, D. *et al.* Landscape preservation under Fennoscandian ice sheets determined from in situ produced Be-10 and Al-26. *Earth and Planetary Science Letters* **201**, 397-406 (2002).

40 Pedro, J. B. *et al.* The spatial extent and dynamics of the Antarctic Cold Reversal. *Nature Geosci*, **9**, 51-55 (2015).

41 Le Maitre, R. A Classification of Igneous Rocks and Glossary of terms. *Blackwell Scientific Publications.* (1989.).

42 Kuehn, SC, Froese, DG, Shane, PAR 2011. The INTAV intercomparison of electron-beam microanalysis of glass by tephrochronology laboratories: Results and recommendations. Quaternary International **246**: 19-47.

43. Wilson, S.A., 1997 The collection, preparation, and testing of USGS reference material BCR-2, Columbia River, Basalt: U.S. Geological Survey Open-File Report
